# An Intelligent Multiattribute Decision-Support Framework Based on Parameterization of Neutrosophic Hypersoft Set

**DOI:** 10.1155/2022/6229947

**Published:** 2022-03-24

**Authors:** Atiqe Ur Rahman, Muhammad Saeed, Alhanouf Alburaikan, Hamiden Abd El-Wahed Khalifa

**Affiliations:** ^1^Department of Mathematics, University of Management and Technology, Lahore 54000, Pakistan; ^2^Department of Mathematics, College of Science and Arts, Al-Badaya, Qassim University, Buraydah, Saudi Arabia; ^3^Department of Operations Research, Faculty of Graduate Studies for Statistical Research, Cairo University, Giza, Egypt

## Abstract

Hypersoft set is a novel area of interest which is able to tackle the real-world scenarios where classification of parameters into their respective sub-parametric values in the form of overlapping sets is mandatory. It employs a new approximate mapping which considers such sets in the form of sub-parametric tuples as its domain. The existing soft set-like structures are insufficient to tackle such kind of situations. This research intends to establish a novel concept of parameterization of fuzzy set under hypersoft set environment with uncertain components of intuitionistic fuzzy set and neutrosophic set. Two novel structures, i.e., fuzzy parameterized intuitionistic fuzzy hypersoft set (fpifhs-set) and fuzzy parameterized neutrosophic hypersoft set (fpnhs-set), are developed by employing algebraic techniques like theoretic, analytical, pictorial, and algorithmic techniques. After characterizing the elementary properties and set-theoretic operations of fpifhs-set and fpnhs-set, two novel algorithms are proposed to solve real-life decision-making COVID-19 problem. The results of both algorithms are compared with related already established models through certain evaluating features to judge the advantageous aspects of the proposed study. The generalization of the proposed models is discussed by describing some of their particular cases.

## 1. Introduction

The conventional logic is not always significant in real-life state of affairs, where the accessible information is unclear or indefinite. To cope with such sort of circumstances, a specific class of sets recognized as fuzzy set (*f*-set) (initiated by Zadeh [1]) is regarded as apt. In such set, a belonging grade within [0, 1] is assigned to each element of the initial universe. Nevertheless, to manage more complex and uncertain real-life situations, *f*-set was found incapable, and therefore Atanassov [2] established a novel model, i.e., intuitionistic fuzzy set (if-set) which is more effective in tackling vagueness of data. It assigns belonging and nonbelonging grades to each element of the initial universe subject to the condition that their sum lies within [0, 1]. Moreover, *if*-sets are proficient at emulating the available information more precisely and rationally. Many researchers made contributions regarding the extensions of *f*-sets and if-sets, but the contributions of Deli and Keleş [3], Mahmood et al. [4], Ünver et al. [5], and Wang and Garg [6] are more significant. They introduced novel extensions of if-sets and applied them in real-world problems through algorithm-based decision-making techniques. Both *f*-sets and if-sets were observed to be incapable of considering the grade of indeterminacy, so neutrosophic set (*n*-set) was instigated by Smarandache [7] to deal with such kind of limitations. The *n*-set is more proficient at preserving the impreciseness in the contents of perceived data and may assist rough reasoning behavior meticulously. Although the expressive capacity of *n*-set is higher than that of the conventional *f*-set and if-set due to the consideration of a neutral grade, they have reasonably higher computational complication over *f*-set and if-set.

The above described structures showed some drawbacks with respect to the consideration of parameterization tool. To manage such insufficiency, Molodtsov [8] developed soft set (*s*-set) as a new mathematical tool. In *s*-set, an approximate mapping is used to map a set of parameters to power set of universe. In order to enhance the applicability of *s*-set in real-world uncertain scenarios, some of its hybridized structures like fuzzy soft set (*fs*-set) [9, 10], intuitionistic fuzzy soft set (*ifs*-set) [11, 12], and neutrosophic soft set (*ns*-set) [13] were developed. Ali et al. [14], Li [15], Maji et al. [16], Pei and Miao [17], and Sezgin and Atagün [18] discussed the rudiments of *s*-set with numerical examples. Babitha and Sunil [19, 20] introduced the concept of relations, functions, and orders under soft set environment. Broumi et al. [21] and Deli [22] developed hybridized structures of *n*-set with *s*-set with interval settings. They described some of their rudimentary properties and applied different methods for their implementation in certain cases.

In various real-life decision-making situations, it is observed that nonoverlapping sets having sub-parametric values corresponding to parameters are required to be considered, but the *s*-set and its hybrids are not projected for such situations. Therefore, Smarandache [23] presented the notion of hypersoft set (*hs*-set) which is capable of handling such situations by using a new approximate mapping having multiargument domain. In order to utilize *hs*-set in other fields of study, its various basic properties, relations, and matrices have been investigated in [24] with the help of supporting examples. Debnath [25] discussed the decision-making application based on weightage operators of fuzzy hypersoft set. Deli [26] developed a novel hybrid of *hs*-set called “neutrosophic valued n-attribute neutrosophic hypersoft set” which generalizes most of the existing fuzzy set-like structures for dealing with uncertainty, vagueness, and indeterminacy. Kamacı and Saqlain [27] discussed the validity of *hs*-set for the entitlement of multidecisive opinions under expert set environment. Martin and Smarandache [28, 29] discussed the hybridization of hypersoft set with plithogenic set and discussed its applications. Rahman et al. [30–33] studied various notional properties of complex set, convex cum concave sets, and parameterization by using the environment of *hs*-set. They also solved real-world decision-making problems with the help of algorithms based on such hybrids. Saeed et al. [34–36] extended the concept by introducing the mappings and graphs of *hs*-set with indeterminate settings and discussed its implementation in product selection and medical science. Saqlain et al. [37, 38] discussed aggregation operators, distance, and similarity measures of neutrosophic hypersoft sets with application in decision-making. Zulqarnain et al. [39] investigated generalized aggregate operators for neutrosophic hypersoft sets with some results.

### 1.1. Research Gap and Motivation

**Figure 1:**
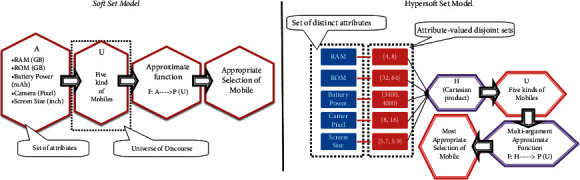
Comparison of soft set model and hypersoft set model.

1. The phrase “parameterization of fuzzy set” is actually meant for dispensing the fuzzy value to each attribute/subattribute in the domain of single-argument/multiargument approximate function (*maa*-function).
2. Many researchers discussed the parameterization of fuzzy set-like structures under soft set environment with fuzzy set-like settings. The literature review of most relevant models is presented in Table 1. It can be observed easily that the above-mentioned models focused on a single set of attributes and used ordinary soft approximate mapping to study the parameterized nature of parametric domain. They are incapable of dealing with situations having compulsion partitioning of attributes into their respective attribute-valued nonoverlapping sets.
3. The incapability of soft set-like models leads to the demand for new structure; therefore, *hs*-set is initiated to manage such situations. As *hs*-set emphasizes deep observation of attributes, *hs*-set can be considered as flexible and reliable model to have unbiased decision-support system. In Figure 1, the difference between *s*-set and *hs*-set is presented with the help of an example for product selection through decision-making.
4. Motivated by the above-mentioned literature in general and [43, 44, 47–50] in specific, we construct novel structures of fuzzy parameterized intuitionistic fuzzy hypersoft set (fpifhs-set) and fuzzy parameterized neutrosophic hypersoft set (fpnhs-set) and characterize them with the help of algorithm-based decision-support systems.

### 1.2. Main Contributions

Main contributions of this research are given below:

1. The structures like those in [43, 44, 47–50] are made capable with the entitlement of *maa*-function by characterizing fpifhs-sets and fpnhs-sets.
2. The situations with sub-parametric-valued sets are managed for the environments of if-set and *n*-set by using fpifhs-sets and fpnhs-sets,
3. Some essential rudiments, i.e., properties, elementary laws, and aggregation operations of fpifhs-set and fpnhs-set are investigated.
4. Two algorithms based on fpifhs-set and fpnhs-set are proposed to deal with daily-life decision-making problems having uncertain data/information in COVID-19 scenario.
5. In order to judge the distinction of this research, the computed algorithm-based results are compared with most relevant models by considering suitable evaluating indicators.
6. The particular cases of the proposed models are discussed along with graphical representation to show their generalized aspects.
7. The paper is summarized with the description of its merits and future scope.

### 1.3. Paper Organization

The rest of the paper is organized as follows: In Section 2, some essential definitions of elementary nature are reviewed from literature for better understanding of the main study. Theories of fpifhs-set and fpnhs-set are developed along with their decision-support systems in Sections 3 and 4, respectively. In order to observe the advantageous aspect of the proposed study, a comparison is presented with some existing relevant models in Section 5, and Section 6 presents the discussion on generalization and merits of proposed work. The paper is summarized with future directions in the last section.

## 2. Preliminaries

This portion of the paper presents some elementary terms and definitions by reviewing the existing literature for clear understanding of the proposed study. Throughout the rest of the paper, the symbols *𝒰*, ℙ(*𝒰*), and *𝕀* denote universe of discourse, power set of *𝒰*, and closed unit interval. Furthermore, *𝔽*(*𝒰*), *𝕀𝔽*(*𝒰*), and *ℕ*(*𝒰*) denote the collection of *f*-sets, if-sets, and *n*-sets over *𝒰*, respectively. In 1965, Zadeh [1] initiated the concept of fuzzy set as a generalization of classical set (crisp set) to deal with uncertain nature of data. This set employs a membership function which maps set of objects (alternatives) to unit closed interval.

**Definition 1 .**

[1].

A fuzzy set *𝒫* is defined as

(1) $$\begin{array}{c} P=\left\{\left(\hat{u},T_{P}\left(\hat{u}\right)\right)|\hat{u}\in U\right\}\text{,} \end{array}$$

such that *T*_*𝒫*_ : *𝒰*⟶*𝕀* where $T_{𝒫}\left(\hat{u}\right)$ represents the membership grade of $\hat{u}\in 𝒫$.

Fuzzy set emphasizes degree of membership only for dealing with uncertain scenarios, but there are many situations where it is necessary to consider nonmembership degree; therefore, into adequate fuzzy set with such situation, Atanassov [2] introduced intuitionistic fuzzy set as a generalization of fuzzy set. It provides due status to both membership and nonmembership degrees of an alternative.

**Definition 2 .**

[2].

An intuitionistic fuzzy set 𝒬 is defined as

(2) $$\begin{array}{c} Q=\left\{\left(\hat{u},<T_{Q}\left(\hat{u}\right),F_{Q}\left(\hat{u}\right)>\right)|\hat{u}\in U\right\}\text{,} \end{array}$$

such that *T*_𝒬_ : *𝒰*⟶*𝕀* and *F*_𝒬_ : *𝒰*⟶*𝕀*, where $T_{𝒬}\left(\hat{u}\right)$ and $F_{𝒬}\left(\hat{u}\right)$ denote the membership and nonmembership grades of $\hat{u}\in 𝒰$ subject to the condition that

(3) $$\begin{array}{c} 0\leq T_{Q}\left(\hat{u}\right)+F_{Q}\left(\hat{u}\right)\leq 1. \end{array}$$

Both fuzzy set and intuitionistic fuzzy set are insufficient to tackle the various uncertain scenarios where the consideration of indeterminate grades is necessary. In order to manage such situations, Smarandache [7] characterized neutrosophic set which not only considers membership and nonmembership grades but also provides due status to degree of indeterminacy of an alternative.

**Definition 3 .**

[7].

A neutrosophic set ℛ is defined as

(4) $$\begin{array}{c} ℛ=\left\{\left(\hat{u},<T_{ℛ}\left(\hat{u}\right),I_{ℛ}\left(\hat{u}\right),F_{ℛ}\left(\hat{u}\right)>\right)|\hat{u}\in U\right\}\text{,} \end{array}$$

such that Tℛu^,Iℛu^,Fℛu^:𝒰⟶0−,1+,where $T_{ℛ}\left(\hat{u}\right),I_{ℛ}\left(\hat{u}\right)$, and $F_{ℛ}\left(\hat{u}\right)$ represent the grades of membership, indeterminacy, and nonmembership of $\hat{u}\in 𝒰$ subject to the condition that

(5) 0−≤Tℛu^+Iℛu^+Fℛu^≤3+.

Fuzzy set, intuitionistic fuzzy set, and neutrosophic set depict some kind of insufficiency regarding the consideration of parameterization tool. In order to manage this limitation, Molodtsov [8] developed soft set as a new mathematical tool to tackle uncertainties and vagueness in the data.

**Definition 4 .**

[8].

A soft set over *𝒰* is a pair (ℱ_*𝒮*_, *𝔾*), where ℱ_*𝒮*_ : *𝔾*⟶ℙ(*𝒰*) and *𝔾*⊆*ℰ* (a set of parameters).

More details on soft set and its operations can be seen in [10, 16]. In many real-world scenarios, the classification of attributes into sub-attributive values in the form of sets is necessary. The existing concept of soft set is not sufficient and is incompatible with such scenarios, so Smarandache [23] introduced hypersoft sets to address the insufficiency of soft set and to handle the situations with multiargument approximate function (MAAF).

**Definition 5 .**

[23].

A hypersoft set over *𝒰* is a pair (*𝒲*, ℋ), where ℋ is the Cartesian product of ℋ^*i*^, *i*=1,2,3,…, *n*, ℋ^*i*^∩ℋ^*j*^=∅ for all *i* ≠ *j* having attribute values of ${\hat{h}}^{i},i=1,2,3,\ldots ,n,{\hat{h}}^{i}\neq {\hat{h}}^{j},i\neq j$, respectively, and *𝒲* : ℋ⟶ℙ(*𝒰*).

For the sake of proper use of hypersoft set under uncertain environments, the hybrids of *s*-sets like *fs*-sets, *ifs*-sets, and *ns*-sets are further modified for hypersoft set environment so the following novel structures are conceptualized.

**Definition 6 .**

[23].

A hypersoft set (*𝒲*, ℋ) is called fuzzy hypersoft set, intuitionistic fuzzy hypersoft set, and neutrosophic hypersoft set if ℙ(*𝒰*) in *𝒲* : ℋ⟶ℙ(*𝒰*) is replaced with *𝔽*(*𝒰*), *𝕀𝔽*(*𝒰*), and *ℕ*(*𝒰*), respectively.

For more details on hypersoft set and its operations, please see [23, 24].

Before presenting main results, first necessity of the proposed study is discussed. In various daily-world scenarios like product selection, supplier selection, and medical diagnosis, the decision-makers face the following problems:

1. The chosen parameters (attributes) need to be further partitioned into their respective attribute-valued disjoint sets.
2. A new type of function (mapping), i.e., multiargument approximated function, is needed to tackle the attribute-valued disjoint sets collectively as its domain.
3. The computed tuples have uncertain nature and need a fuzzy membership value to depict their uncertainty.
4. The approximate elements of multi-approximate function are assessed in the form of intuitionistic fuzzy values or neutrosophic values.

In order to manage all above problems collectively, parameterization of hypersoft set with fuzzy setting is required under intuitionistic fuzzy set and neutrosophic set environments which lead to the demand for the proposed study.

## 3. Fuzzy Parameterized Intuitionistic Fuzzy Hypersoft Set (fpifhs-Set) with Application

**Figure 2:**
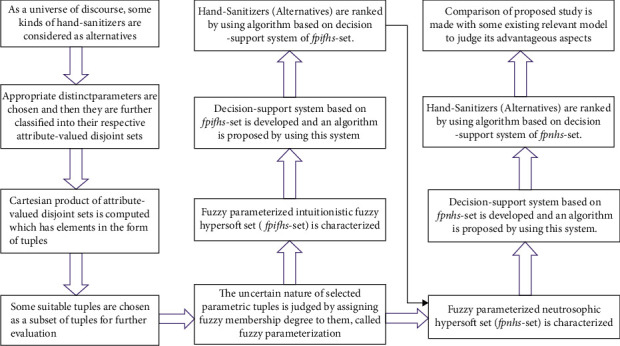
Methodology of the proposed study.

In various real-world decision-making scenarios, the decision-makers are uncertain about the nature of selected parameters under *hs*-set environment with approximate elements having intuitionistic fuzzy values. Such type of parameters is known as fuzzy parameters, and such scenarios are tackled with the help of fpifhs-set. The methodology of the proposed study is explained in Figure 2. In this section, firstly theory of fpifhs-set is constructed, and then decision-making problem is discussed with the help of proposed algorithm.

**Definition 7 .**

Let *𝒳*^1^, *𝒳*^2^, *𝒳*^3^,…, *𝒳*^*n*^ be parameter-valued sets with *𝒳*^*i*^∩*𝒳*^*j*^=∅, *i* ≠ *j* respective to parameters *x*^1^, *x*^2^, *x*^3^,…, *x*^*n*^, *x*^*i*^ ≠ *x*^*j*^, *i* ≠ *j*, respectively. A fpifhs-set Ψ_ℬ_ over *𝒰* is defined as

(6) $$\begin{array}{c} \Psi_{ℬ}=\begin{array}{c} \left\{\left(\frac{L_{ℬ}\left(\hat{g}\right)}{\hat{g}},\psi_{ℬ}\left(\hat{g}\right)\right):\hat{g}\in G,\psi_{ℬ}\left(\hat{g}\right)\in IF\left(U\right)\right\} \end{array}, \end{array}$$

where

- *𝔾*=*𝒳*^1^ × *𝒳*^2^ × *𝒳*^3^ × ….×*𝒳*^*n*^.
- ℬ ∈ *𝔽*(*𝒰*) with *L*_ℬ_ : *𝔾*⟶*𝕀*.
- *ψ*_ℬ_ : *𝔾*⟶*𝕀𝔽*(*𝒰*) is known as approximate function of fpifhs-set.

**Figure 3:**
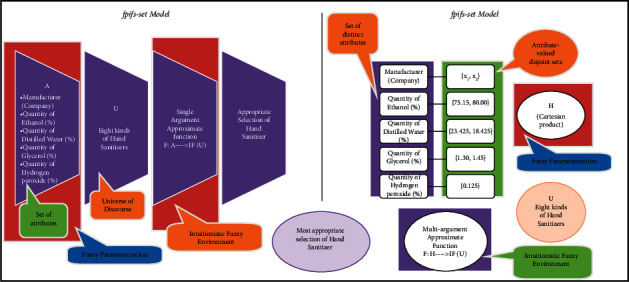
Comparison of fpifs-set model and fpifhs-set model with the help of Example 2.

Note that collection of all fpifhs-sets is represented by $\underset{\text{fpifhs}}{⊎}\left(𝒰\right)$. The vivid comparison of the existing model fpifs-set and the proposed fpifhs-set model with the help of Example 2 can be viewed in Figure 3.

**Definition 8 .**

Let $\Psi_{ℬ}\in \underset{\text{fpifhs}}{⊎}\left(𝒰\right)$. If $\psi_{ℬ}\left(\hat{g}\right)=\emptyset ,L_{ℬ}\left(\hat{g}\right)=0$ for all $\hat{g}\in 𝔾$, then Ψ_ℬ_ is called ℬ-empty fpifhs-set, denoted by Ψ_Φ_ℬ__. If ℬ=∅, then ℬ-empty fpifhs-set is called an empty fpifhs-set, denoted by Ψ_Φ_.

**Definition 9 .**

Let $\Psi_{ℬ}\in \underset{\text{fpifhs}}{⊎}\left(𝒰\right)$. If $\psi_{ℬ}\left(\hat{g}\right)=𝒰,L_{ℬ}\left(\hat{g}\right)=1$ for all $\hat{g}\in 𝔾$, then Ψ_ℬ_ is called ℬ-universal fpifhs-set, denoted by $\Psi_{\tilde{ℬ}}$. If ℬ=*𝔾*, then the ℬ-universal fpifhs-set is called universal fpifhs-set, denoted by $\Psi_{\tilde{𝔾}}$.

**Example 1 .**

Consider

$𝒰=\left\{{\hat{u}}_{1},{\hat{u}}_{2},{\hat{u}}_{3},{\hat{u}}_{4},{\hat{u}}_{5}\right\}$ and *𝒴*={*𝒴*_1_, *𝒴*_2_, *𝒴*_3_} with ${𝒴}_{1}=\left\{{\hat{y}}_{11},{\hat{y}}_{12}\right\}$, ${𝒴}_{2}=\left\{{\hat{y}}_{21},{\hat{y}}_{22}\right\},{𝒴}_{3}=\left\{{\hat{y}}_{31}\right\}$; then, *𝔾*=*𝒴*_1_ × *𝒴*_2_ × *𝒴*_3_, $𝔾=\left\{\begin{array}{c} \left({\hat{y}}_{11},{\hat{y}}_{21},{\hat{y}}_{31}\right),\left({\hat{y}}_{11},{\hat{y}}_{22},{\hat{y}}_{31}\right),\\ \left({\hat{y}}_{12},{\hat{y}}_{21},{\hat{y}}_{31}\right),\left({\hat{y}}_{12},{\hat{y}}_{22},{\hat{y}}_{31}\right) \end{array}\right\}𝔾=\left\{{\hat{g}}_{1},{\hat{g}}_{2},{\hat{g}}_{3},{\hat{g}}_{4}\right\}$.

  Case 1. When ${ℬ}_{1}=\left\{0.2/{\hat{g}}_{2},0.0/{\hat{g}}_{3},1.0/{\hat{g}}_{4}\right\}$, $\psi_{{ℬ}_{1}}\left({\hat{g}}_{2}\right)=\left\{\left(0.2,0.4\right)/{\hat{u}}_{2},\left(0.3,0.5\right)/{\hat{u}}_{4}\right\}$, $\psi_{{ℬ}_{1}}\left({\hat{g}}_{3}\right)=\emptyset$, and $\psi_{{ℬ}_{1}}\left({\hat{g}}_{4}\right)=𝒰$, then $\Psi_{{ℬ}_{1}}=\left\{\begin{array}{c} \left(0.2/{\hat{g}}_{2},\left\{\begin{array}{c} \left(0.2,0.4\right)/{\hat{u}}_{2},\\ \left(0.3,0.5\right)/{\hat{u}}_{4} \end{array}\right\}\right),\\ \left(0.0/{\hat{g}}_{3},\emptyset \right),\left(1.0/{\hat{g}}_{4},𝒰\right) \end{array}\right\}$.
  Case 2. When ${ℬ}_{2}=\left\{0.0/{\hat{g}}_{2},0.0/{\hat{g}}_{3}\right\},\psi_{{ℬ}_{2}}\left({\hat{g}}_{2}\right)=\emptyset$ and $\psi_{{ℬ}_{2}}\left({\hat{g}}_{3}\right)=\emptyset$, then Ψ_ℬ_2__=Ψ_Φ_ℬ_2___.
  Case 3. When ℬ_3_=∅ for all elements of *𝔾*, then Ψ_ℬ_3__=Ψ_Φ_.
  Case 4. When ${ℬ}_{4}=\left\{1.0/{\hat{g}}_{1},1.0/{\hat{g}}_{2}\right\},\psi_{{ℬ}_{4}}\left({\hat{g}}_{1}\right)=𝒰$, and $\psi_{{ℬ}_{4}}\left({\hat{g}}_{2}\right)=𝒰$, then $\Psi_{{ℬ}_{4}}=\Psi_{\tilde{{ℬ}_{4}}}$.
  Case 5. When ℬ_5_=*𝒰* for all elements of *𝔾*, then $\Psi_{{ℬ}_{5}}=\Psi_{\tilde{𝔾}}$.

**Definition 10 .**

Let Ψ_ℬ_1__, $\Psi_{{ℬ}_{2}}\in \underset{\text{fpifhs}}{⊎}\left(𝒰\right)$; then, Ψ_ℬ_1__ is an fpifhs-subset of Ψ_ℬ_2__, denoted by $\Psi_{{ℬ}_{1}}{\tilde{\subseteq }}_{f}\Psi_{{ℬ}_{2}}$ if$L_{{ℬ}_{1}}\left(\hat{g}\right)\leq L_{{ℬ}_{2}}\left(\hat{g}\right)$ and $\psi_{{ℬ}_{1}}\left(\hat{g}\right)\subseteq_{if}\psi_{{ℬ}_{2}}\left(\hat{g}\right)$ for all $\hat{g}\in 𝔾$. Here, ${\tilde{\subseteq }}_{f}$ and ⊆_if_ represent fuzzy subset and intuitionistic fuzzy subset notations, respectively.

**Definition 11 .**

Let $\Psi_{{ℬ}_{1}},\Psi_{{ℬ}_{2}}\in \underset{\text{fpifhs}}{⊎}\left(𝒰\right)$; then, Ψ_ℬ_1__ and Ψ_ℬ_2__ are fpifhs-equal, represented as Ψ_ℬ_1__=Ψ_ℬ_2__, if and only if $L_{{ℬ}_{1}}\left(\hat{g}\right)=L_{{ℬ}_{2}}\left(\hat{g}\right)$ and $\psi_{{ℬ}_{1}}\left(\hat{g}\right){=}_{\text{if}}\psi_{{ℬ}_{2}}\left(\hat{g}\right)$ for all $\hat{g}\in 𝔾$.

**Definition 12 .**

Let $\Psi_{ℬ}\in \underset{\text{fpifhs}}{⊎}\left(𝒰\right)$; then, complement of Ψ_ℬ_ (i.e., $\Psi_{ℬ}^{\tilde{c}}$) is an fpifhs-set given as $P_{ℬ}^{\tilde{c}}\left(\hat{g}\right)=1-L_{ℬ}\left(\hat{g}\right)$ and $\psi_{ℬ}^{\tilde{c}}\left(\hat{g}\right)=𝒰\setminus_{if}\psi_{ℬ}\left(\hat{g}\right)$.

**Proposition 1 .**

Let $\Psi_{ℬ}\in \underset{\text{fpifhs}}{⊎}\left(𝒰\right)$; then,

1. ${\left(\Psi_{ℬ}^{\tilde{c}}\right)}^{\tilde{c}}=\Psi_{ℬ}$.
2. $\Psi_{\phi }^{\tilde{c}}=\Psi_{\tilde{𝔾}}$.

**Definition 13 .**

Let $\Psi_{{ℬ}_{1}},\Psi_{{ℬ}_{2}}\in \underset{\text{fpifhs}}{⊎}\left(𝒰\right)$; then, union of Ψ_ℬ_1__ and Ψ_ℬ_2__, denoted by $\Psi_{{ℬ}_{1}}{\tilde{\cup }}_{f}\Psi_{{ℬ}_{2}}$, is a fpifhs-set defined by

- $L_{{ℬ}_{1}\tilde{\cup }{ℬ}_{2}}\left(\hat{g}\right)=\max\left\{L_{{ℬ}_{1}}\left(x\right),L_{{ℬ}_{2}}\left(\hat{g}\right)\right\}$.
- $\psi_{{ℬ}_{1}\tilde{\cup }{ℬ}_{2}}\left(\hat{g}\right)=\psi_{{ℬ}_{1}}\left(\hat{g}\right)\cup_{if}\psi_{{ℬ}_{2}}\left(\hat{g}\right)$, for all $\hat{g}\in 𝔾$.

**Definition 14 .**

Let $\Psi_{{ℬ}_{1}},\Psi_{{ℬ}_{2}}\in \underset{\text{fpifhs}}{⊎}\left(𝒰\right)$; then, intersection of Ψ_ℬ_1__ and Ψ_ℬ_2__, denoted by $\Psi_{{ℬ}_{1}}{\tilde{\cap }}_{f}\Psi_{{ℬ}_{2}}$, is a fpifhs-set defined by

- $L_{{ℬ}_{1}\tilde{\cap }{ℬ}_{2}}\left(\hat{g}\right)=\min\left\{L_{{ℬ}_{1}}\left(x\right),L_{{ℬ}_{2}}\left(\hat{g}\right)\right\}$.
- $\psi_{{ℬ}_{1}\tilde{\cap }{ℬ}_{2}}\left(\hat{g}\right)=\psi_{{ℬ}_{1}}\left(\hat{g}\right)\cap_{\text{if}}\psi_{{ℬ}_{2}}\left(\hat{g}\right)$, for all $\hat{g}\in 𝔾$.

**Remark 1 .**

For $\Psi_{ℬ}\in \underset{\text{fpifhs}}{⊎}\left(𝒰\right)$. If $\Psi_{ℬ}\neq_{f}\Psi_{\tilde{𝔾}}$, then $\Psi_{ℬ}{\tilde{\cup }}_{f}\Psi_{ℬ}^{\tilde{c}}\neq_{f}\Psi_{\tilde{𝔾}}$ and $\Psi_{ℬ}{\tilde{\cap }}_{f}\Psi_{ℬ}^{\tilde{c}}\neq_{f}\Psi_{\Phi }$.

**Proposition 2 .**

Let $\Psi_{{ℬ}_{1}},\Psi_{{ℬ}_{2}}\in \underset{\text{fpifhs}}{⊎}\left(𝒰\right)$; then, the following De Morgan laws are valid:

1. ${\left(\Psi_{{ℬ}_{1}}{\tilde{\cup }}_{f}\Psi_{{ℬ}_{2}}\right)}^{\tilde{c}}=\Psi_{{ℬ}_{1}}^{\tilde{c}}{\tilde{\cap }}_{f}\Psi_{{ℬ}_{2}}^{\tilde{c}}$.
2. ${\left(\Psi_{{ℬ}_{1}}{\tilde{\cap }}_{f}\Psi_{{ℬ}_{2}}\right)}^{\tilde{c}}=\Psi_{{ℬ}_{1}}^{\tilde{c}}{\tilde{\cup }}_{f}\Psi_{{ℬ}_{2}}^{\tilde{c}}$.

**Proof**

For all $\hat{g}\in 𝔾$:

(1) Since
  (7) $$\begin{array}{c} {\left(L_{{ℬ}_{1}\tilde{\cup }{ℬ}_{2}}\right)}^{\tilde{c}}\left(\hat{g}\right)=1-L_{{ℬ}_{1}\tilde{\cup }{ℬ}_{2}}\left(\hat{g}\right)\\ =1-\max\left\{L_{{ℬ}_{1}}\left(\hat{g}\right),L_{{ℬ}_{2}}\left(\hat{g}\right)\right\}\\ =\min\left\{1-L_{{ℬ}_{1}}\left(\hat{g}\right),1-L_{{ℬ}_{2}}\left(\hat{g}\right)\right\}\\ =\min\left\{P_{{ℬ}_{1}}^{\tilde{c}}\left(\hat{g}\right),P_{{ℬ}_{2}}^{\tilde{c}}\left(\hat{g}\right)\right\}\\ =P_{{ℬ}_{1}\tilde{\cap }{ℬ}_{2}}^{\tilde{c}}\left(\hat{g}\right), \end{array}$$
and
  (8) $$\begin{array}{c} {\left(\psi_{{ℬ}_{1}\tilde{\cup }{ℬ}_{2}}\right)}^{\tilde{c}}\left(\hat{g}\right)=U\setminus_{\text{if}}\psi_{{ℬ}_{1}\tilde{\cup }{ℬ}_{2}}\left(\hat{g}\right)\\ =U\setminus_{\text{if}}\left(\psi_{{ℬ}_{1}}\left(\hat{g}\right)\cup_{\text{if}}\psi_{{ℬ}_{2}}\left(\hat{g}\right)\right)\\ =\left(U\setminus_{\text{if}}\psi_{{ℬ}_{1}}\left(\hat{g}\right)\right)\cap_{\text{if}}\left(U\setminus_{\text{if}}\psi_{{ℬ}_{2}}\left(\hat{g}\right)\right)\\ =\psi_{{ℬ}_{1}}^{\tilde{c}}\left(\hat{g}\right)\tilde{\cap_{\text{if}}}\psi_{{ℬ}_{2}}^{\tilde{c}}\left(\hat{g}\right)\\ =\psi_{{ℬ}_{1}\tilde{\cap }{ℬ}_{2}}^{\tilde{c}}\left(\hat{g}\right)\text{,} \end{array}$$

similarly, (2) can be proved easily.

**Proposition 3 .**

Let $\Psi_{{ℬ}_{1}},\Psi_{{ℬ}_{2}},\Psi_{{ℬ}_{3}}\in \underset{\text{fpifhs}}{⊎}\left(𝒰\right)$; then,

1. $\Psi_{{ℬ}_{1}}{\tilde{\cup }}_{f}\left(\Psi_{{ℬ}_{2}}{\tilde{\cap }}_{f}\Psi_{{ℬ}_{3}}\right)=\;\left(\Psi_{{ℬ}_{1}}{\tilde{\cup }}_{f}\Psi_{{ℬ}_{2}}\right){\tilde{\cap }}_{f}\left(\Psi_{{ℬ}_{1}}{\tilde{\cup }}_{f}\Psi_{{ℬ}_{3}}\right)$.
2. $\Psi_{{ℬ}_{1}}{\tilde{\cap }}_{f}\left(\Psi_{{ℬ}_{2}}{\tilde{\cup }}_{f}\Psi_{{ℬ}_{3}}\right)=\;\left(\Psi_{{ℬ}_{1}}{\tilde{\cap }}_{f}\Psi_{{ℬ}_{2}}\right){\tilde{\cup }}_{f}\left(\Psi_{{ℬ}_{1}}{\tilde{\cap }}_{f}\Psi_{{ℬ}_{3}}\right)$.

**Proof**

For all $\hat{g}\in 𝔾$:

(1) Since
  (9) $$\begin{array}{c} L_{{ℬ}_{1}\tilde{\cup }\left({ℬ}_{2}\tilde{\cap }{ℬ}_{3}\right)}\left(\hat{g}\right)=\max\left\{L_{{ℬ}_{1}}\left(\hat{g}\right),L_{{ℬ}_{2}\tilde{\cap }{ℬ}_{3}}\left(\hat{g}\right)\right\}\\ =\max\left\{L_{{ℬ}_{1}}\left(\hat{g}\right),\min\left\{L_{{ℬ}_{2}}\left(\hat{g}\right),L_{{ℬ}_{3}}\left(\hat{g}\right)\right\}\right\}\\ =\min\left\{\max\left\{L_{{ℬ}_{1}}\left(\hat{g}\right),L_{{ℬ}_{2}}\right\}\left(\hat{g}\right),\max\left\{L_{{ℬ}_{1}}\left(\hat{g}\right),L_{{ℬ}_{3}}\left(\hat{g}\right)\right\}\right\}\\ =\min\left\{L_{{ℬ}_{1}\tilde{\cup }{ℬ}_{2}}\left(\hat{g}\right),L_{{ℬ}_{1}\tilde{\cup }{ℬ}_{3}}\left(\hat{g}\right)\right\}\\ =L_{\left({ℬ}_{1}\tilde{\cup }{ℬ}_{2}\right)\tilde{\cap }\left({ℬ}_{1}\tilde{\cup }{ℬ}_{3}\right)}\left(\hat{g}\right), \end{array}$$
and
  (10) $$\begin{array}{c} \psi_{{ℬ}_{1}\tilde{\cup }\left({ℬ}_{2}\tilde{\cap }{ℬ}_{3}\right)}\left(\hat{g}\right)=\psi_{{ℬ}_{1}}\left(\hat{g}\right)\cup_{\text{if}}\psi_{{ℬ}_{2}\tilde{\cap }{ℬ}_{3}}\left(\hat{g}\right)\\ =\psi_{{ℬ}_{1}}\left(\hat{g}\right)\cup_{if}\left(\psi_{{ℬ}_{2}}\left(\hat{g}\right)\cap_{if}\psi_{{ℬ}_{3}}\left(\hat{g}\right)\right)\\ =\left(\psi_{{ℬ}_{1}}\left(\hat{g}\right)\cup_{\text{if}}\psi_{{ℬ}_{2}}\left(\hat{g}\right)\right)\cap_{\text{if}}\left(\psi_{{ℬ}_{1}}\left(\hat{g}\right)\cup_{\text{if}}\psi_{{ℬ}_{3}}\left(\hat{g}\right)\right)\\ =\psi_{{ℬ}_{1}\tilde{\cup }{ℬ}_{2}}\left(\hat{g}\right)\cap_{\text{if}}\psi_{{ℬ}_{1}\tilde{U}{ℬ}_{3}}\left(\hat{g}\right)\\ =\psi_{\left({ℬ}_{1}\tilde{\cup }{ℬ}_{2}\right)\tilde{\cap }\left({ℬ}_{1}\tilde{\cup }{ℬ}_{3}\right)}\left(\hat{g}\right)\text{,} \end{array}$$

in the same way, (2) can be proved.

**Definition 15 .**

Let $\Psi_{{ℬ}_{1}},\Psi_{{ℬ}_{2}}\in \underset{\text{fpifhs}}{⊎}\left(𝒰\right)$; then, OR-operation of Ψ_ℬ_1__ and Ψ_ℬ_2__, denoted by $\Psi_{{ℬ}_{1}}\tilde{⊻}\Psi_{{ℬ}_{2}}$, is an fpifhs-set defined by

- $L_{{ℬ}_{1}\tilde{⊻}{ℬ}_{2}}\left({\hat{g}}_{1},{\hat{g}}_{2}\right)=\max\left\{L_{{ℬ}_{1}}\left({\hat{g}}_{1}\right),L_{{ℬ}_{2}}\left({\hat{g}}_{2}\right)\right\}$,
- $\psi_{{ℬ}_{1}\tilde{⊻}{ℬ}_{2}}\left({\hat{g}}_{1},{\hat{g}}_{2}\right)=\psi_{{ℬ}_{1}}\left({\hat{g}}_{1}\right)\cup_{\text{if}}\psi_{{ℬ}_{2}}\left({\hat{g}}_{2}\right)$, for all $\left({\hat{g}}_{1},{\hat{g}}_{2}\right)\in {ℬ}_{1}\times {ℬ}_{2}$.

**Definition 16 .**

Let $\Psi_{{ℬ}_{1}},\Psi_{{ℬ}_{2}}\in \underset{\text{fpifhs}}{⊎}\left(𝒰\right)$; then, AND-operation of Ψ_ℬ_1__ and Ψ_ℬ_2__, denoted by $\Psi_{{ℬ}_{1}}\tilde{⊼}\Psi_{{ℬ}_{2}}$, is an fpifhs-set defined by

- $L_{{ℬ}_{1}\tilde{⊼}{ℬ}_{2}}\left({\hat{g}}_{1},{\hat{g}}_{2}\right)=\min\left\{L_{{ℬ}_{1}}\left({\hat{g}}_{1}\right),L_{{ℬ}_{2}}\left({\hat{g}}_{2}\right)\right\}$.
- $\psi_{{ℬ}_{1}\tilde{⊼}{ℬ}_{2}}\left({\hat{g}}_{1},{\hat{g}}_{2}\right)=\psi_{{ℬ}_{1}}\left({\hat{g}}_{1}\right)\cap_{if}\psi_{{ℬ}_{2}}\left({\hat{g}}_{2}\right)$, for all $\left({\hat{g}}_{1},{\hat{g}}_{2}\right)\in {ℬ}_{1}\times {ℬ}_{2}$.

**Proposition 4 .**

Let $\Psi_{{ℬ}_{1}},\Psi_{{ℬ}_{2}},\Psi_{{ℬ}_{3}}\in \underset{\text{fpifhs}}{⊎}\left(𝒰\right)$; then,

1. $\Psi_{{ℬ}_{1}}\tilde{⊼}\Psi_{\Phi }=\Psi_{\Phi }$.
2. $\left(\Psi_{{ℬ}_{1}}\tilde{⊼}\Psi_{{ℬ}_{2}}\right)\tilde{⊼}\Psi_{{ℬ}_{3}}=\Psi_{{ℬ}_{1}}\tilde{⊼}\left(\Psi_{{ℬ}_{2}}\tilde{⊼}\Psi_{{ℬ}_{3}}\right)$.
3. $\left(\Psi_{{ℬ}_{1}}\tilde{⊻}\Psi_{{ℬ}_{2}}\right)\tilde{⊻}\Psi_{{ℬ}_{3}}=\Psi_{{ℬ}_{1}}\tilde{⊻}\left(\Psi_{{ℬ}_{2}}\tilde{⊻}\Psi_{{ℬ}_{3}}\right)$.

### 3.1. Fuzzy Decision Set of fpifhs-Set

In this part, fuzzy decision-support system based on fpifhs-set will be established.

**Definition 17 .**

Let $\Psi_{ℬ}\in \underset{\text{fpifhs}}{⊎}\left(𝒰\right)$; then, a fuzzy decision set of Ψ_ℬ_ (i.e., Ψ_ℬ_^*D*^) is

represented as

(11) $$\begin{array}{c} \Psi_{ℬ}^{D}=\left\{\frac{T_{ℬ}^{D}\left(\hat{u}\right)}{\hat{u}:\hat{u}\in U}\right\}, \end{array}$$

where *𝒯*_ℬ_^*D*^ : *𝒰*⟶*𝕀* and

(12) $$\begin{array}{c} T_{ℬ}^{D}\left(\hat{u}\right)=\frac{1}{\left|U\right|}\sum_{v\in S\left(ℬ\right)}T_{ℬ}\left(v\right)\Gamma_{\psi_{ℬ}\left(v\right)}\left(\hat{u}\right), \end{array}$$

where |•| denotes set cardinality with

(13) $$\begin{array}{c} \Gamma_{\psi_{ℬ}\left(v\right)}\left(\hat{u}\right)=\left\{\begin{array}{cc} \left|T_{\psi_{ℬ}}\left(\hat{u}\right)-F_{\psi_{ℬ}}\left(\hat{u}\right)\right|, & \hat{u}\in \Gamma_{\psi_{ℬ}}\left(v\right),\\ 0, & \hat{u}\notin \Gamma_{\psi_{ℬ}}\left(v\right). \end{array}\right. \end{array}$$

With the establishment of Ψ_ℬ_^*D*^, optimal single selection from the set of alternatives can easily be evalauated. Therefore, the following algorithm is proposed to make appropriate decision.

**Figure 4:**
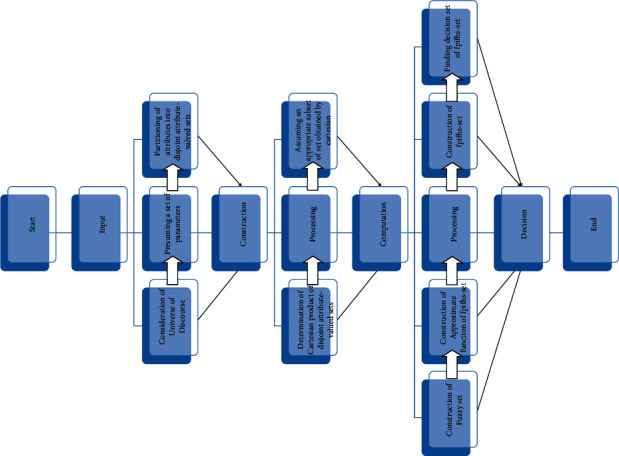
Decision-making algorithm for fpifhs-set.

Figure 4 presents the flowchart of Algorithm 1.

#### 3.1.1. Problem Scenario

**Figure 5:**
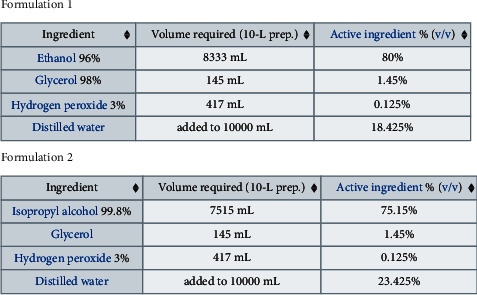
Formulations of hand sanitizers, figure source: Wikipedia (https://en.wikipedia.org/wiki/Hand_sanitizer).

Hand sanitizer is a fluid or gel that is utilized to kill germs on the hands. As per the World Health Organization (WHO), excellent sterilization and actual detachment are simply the most ideal approaches to rescue the individuals from COVID-19 in the ebb and flow illness circumstance. This infection is communicated by reaching somebody who is debilitated. We cannot be excessively cautious with this infection on the off chance that we do not isolate ourselves totally. Accordingly, great disinfection can be the best way to guard us against the infection. WHO suggests alcohol-based hand sanitizers (ABH-sanitizers) for eliminating the novel corona virus. ABH-sanitizers prevent germ proteins, like microscopic organisms and infections, from working ordinarily. There are two kinds of formulations (see Figure 5) recommended by WHO for the production of large quantities of hand sanitizer from chemicals available in developing countries, where commercial hand sanitizer may not be available. The interest in hand sanitizers has expanded drastically in light of COVID-19's critical situation. Thus, discovering great and powerful hand sanitizers in local markets is troublesome. Because of the expanded interest, inferior quality hand sanitizers have additionally been dispatched. The fundamental objective of this application is to utilize fpifhs-set and fpnhs-set theories to track down a successful sanitizer to stop the spread of COVID-19.

#### 3.1.2. Operational Role of Constituents of ABH-Sanitizers

It is clear from Figure 5 that ABH-sanitizers have four major constituents that are given below with their brief operation role in sanitation process:

1. Ethanol: It is an organic chemical compound and majorly used in medication as antiseptic, antidote, and medicinal solvent. It is the major constituent of ABH-sanitizers, and it ranges from 70% to 80%(*V*/*V*) as an active ingredient.
2. Glycerol: It is a nontoxic viscous liquid having no color, no odor, and sweet taste. It is mildly antimicrobial and antiviral. It prevents skin dryness with its moisturizing properties. It draws moisture up through skin layers and slows or prevents excessive drying and evaporation. It is added to ABH-sanitizers to prevent drying of the skin, and it ranges from 1.35% to 1.45%(*V*/*V*) as an active ingredient.
3. Hydrogen peroxide: It is a very pale blue colored chemical compound having viscosity slightly more than water. Usually as a dilute solution (3–6% by weight) in water, it is used as an oxidizer, bleaching agent, and antiseptic for consumer use. It can be used for industrial purposes with high concentrations. It is used in ABH-sanitizers as sterilizer, and it ranges from 0.120% to 0.125%(*V*/*V*) as an active ingredient.
4. Distilled water: It is an essential ingredient of ABH-sanitizers. It ranges from 15.425 % to 23.425%(*V*/*V*) as an active ingredient. It is used as cleansing agent to prevent the interaction of chlorine and other contaminants with the alcohol and other ingredients in the sanitizer.

#### 3.1.3. Statement of the Problem

Professor John is the head of an educational institution. He is very concerned about the health of the students as well as the faculty members of his institution in view of the current coronary epidemic. He wants to buy good and useful hand sanitizers for his institution, but he is also worried about the nonstandard hand sanitizers available in the market. Therefore, he decides to call for bids from different potential suppliers for this purchase to fulfill the departmental official compliances and to avoid any expected loss. Some suppliers are scrutinized by adopting proper procedure already framed by relevant department. For the sake of satisfaction, he constitutes a committee consisting of some staff members with good procurement experience to evaluate the items (hand sanitizers) offered by scrutinized suppliers. The following example elaborates the whole procedure of such evaluation:

**Example 2 .**

**Figure 6:**
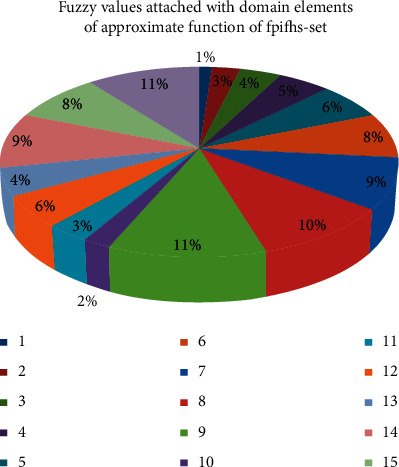
Graphical representation of Table 2.

**Figure 7:**
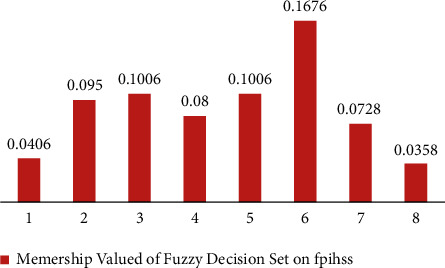
Fuzzy decision system on fpifhs-set.

Input and Construction Stages (1–5):
Suppose there are eight kinds of hand sanitizer (options) which form the set of discourse
  (14) $$\begin{array}{c} U=\left\{U_{1},U_{2},U_{3}\right\}, \end{array}$$
where *𝒰*_1_={ℍ^1^, ℍ^2^, ℍ^3^}, *𝒰*_2_={ℍ^4^, ℍ^5^, ℍ^6^}, and *𝒰*_3_={ℍ^7^, ℍ^8^} are the collection of hand sanitizers made by manufacturers *X*_1_, *X*_2_, and *X*_3_, respectively. With their mutual consensus, the committee members (experts) agreed on a set of parameters after observing various attributes for this evaluation. The finalized evaluating attributes are *b*^1^  manufacturer; *b*^2^  quantity of ethanol (percentage); *b*^3^  quantity of distilled water (percentage); *b*^4^  quantity of glycerol (percentage); and *b*^5^  quantity of hydrogen peroxide (percentage). After observing the opinions of various professionals and other relevant sources on the composition of hand sanitizers, these attributes are further classified into attribute-valued sets which are given as
  (15) $$\begin{array}{c} B^{1}=\left\{b^{11}=X_{1},b^{12}=X_{2},b^{13}=X_{3}\right\}\text{,}\\ B^{2}=\left\{b^{21}=75.15,b^{22}=80\right\}\text{,}\\ B^{3}=\left\{b^{31}=23.425,b^{32}=18.425\right\}\text{,}\\ B^{4}=\left\{b^{41}=1.30,b^{42}=1.45\right\}\text{,}\\ B^{5}=\left\{b^{51}=0.125\right\}. \end{array}$$
Then, *ℚ*=*B*^1^ × *B*^2^ × *B*^3^ × *B*^4^ × *B*^5^,
*ℚ*={*q*^1^, *q*^2^, *q*^3^, *q*^4^,….., *q*^24^}, where each *q*^*i*^, *i*=1,2,…, 24, is a 5-tuple element. For convenience, take *ℝ*={*q*^1^, *q*^2^, *q*^3^, *q*^4^,….., *q*}^16^⊆*ℚ*.
Computation Stage (6–9):
(6) The graphical depiction of Table 2 can be seen in Figure 6.
From Table 2, we can construct ℬ as
  (16) $$\begin{array}{c} ℬ=\left\{\begin{array}{c} \frac{0.1}{q^{1}},\frac{0.2}{q^{2}},\frac{0.3}{q^{3}},\frac{0.4}{q^{4}},\\ \frac{0.5}{q^{5}},\frac{0.6}{q^{6}},\frac{0.7}{q^{7}},\frac{0.8}{q^{8}},\\ \frac{0.9}{q^{9}},\frac{0.16}{q^{10}}\frac{0.25}{q^{11}}\frac{0.45}{q^{12}},\\ \frac{0.35}{q^{13}},\frac{0.75}{q^{14}},\frac{0.65}{q^{15}},\frac{0.85}{q^{16}} \end{array}\right\}. \end{array}$$
(7) Table 3 presents *ψ*_ℬ_(*q*^*i*^) corresponding to each element of *𝔾*.
(8) Ψ_ℬ_ can be constructed with the support of step 6 and step 7 as performed previously.
(9) From Table 4, we can construct Ψ_ℬ_^*D*^ as
  (17) $$\begin{array}{c} \Psi_{ℬ}^{D}=\left\{\begin{array}{c} \frac{0.0406}{{ℍ}^{1}},\frac{0.0950}{{ℍ}^{2}},\frac{0.1006}{{ℍ}^{3}},\frac{0.0800}{{ℍ}^{4}},\\ \frac{0.1006}{{ℍ}^{5}},\frac{0.1676}{{ℍ}^{6}},\frac{0.0728}{{ℍ}^{7}},\frac{0.0358}{{ℍ}^{8}} \end{array}\right\}. \end{array}$$
For its pictorial representation, please see Figure 7.
Output Stage:
(10) Since maximum of *𝒯*_ℬ_^*D*^(ℍ^*i*^) is 0.1676, hand sanitizer ℍ^6^ is selected.

## 4. Fuzzy Parameterized Neutrosophic Hypersoft Set (fpnhs-Set) with Application

fpifhs-set is quite better to tackle real-world decision-making scenarios where the decision-makers are uncertain about the nature of selected parameters under *hs*-set environment with approximate elements having intuitionistic fuzzy values. However, it is still insufficient to manage the situation when approximate elements are needed to be assessed with indeterminacy degree; therefore, this shortcoming demands another structure to deal with these situations. Therefore, in this section, the theory of fpnhs-set is developed with the characterization of some of its essential rudiments and decision-support system.

**Definition 18 .**

Let *𝒮*^1^, *𝒮*^2^, *𝒮*^3^,…., *𝒮*^*n*^ be parameter-valued sets with *𝒮*^*i*^∩*𝒮*^*j*^=∅, *i* ≠ *j* respective to parameters*s*^1^, *s*^2^, *s*^3^,…, *s*^*n*^, *s*^*i*^ ≠ *s*^*j*^, *i* ≠ *j*, respectively. A fpnhs-set Ψ_*𝒟*_ over *𝒰* is defined as

(18) $$\begin{array}{c} \Psi_{D}=\left\{\begin{array}{c} \left(\frac{A_{D}\left(\hat{g}\right)}{\hat{g}},\psi_{D}\left(\hat{g}\right)\right):\hat{g}\in G,\psi_{D}\left(\hat{g}\right)\in \mathbb{N}\left(U\right) \end{array}\right\}, \end{array}$$

where

- *𝔾*=*𝒮*^1^ × *𝒮*^2^ × *𝒮*^3^ × ….×*𝒮*^*n*^.
- *𝒟* ∈ *𝔽*(*𝒰*) with *L*_*𝒟*_ : *𝔾*⟶*𝕀*.
- *ψ*_*𝒟*_ : *𝔾*⟶*ℕ*(*𝒰*) is known as approximate function of fpnhs-set.

**Figure 8:**
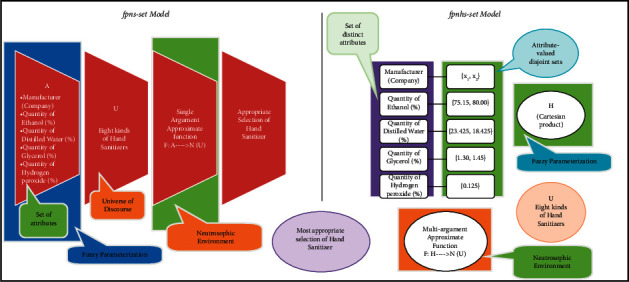
Comparison of fpns-set model and fpnhs-set model with the help of Example 4.

Note that collection of all fpnhs-sets is represented by $\underset{\text{fpnhs}}{⊎}\left(𝒰\right)$. The pictorial self-explanatory comparison of the existing model fpns-set and the proposed fpnhs-set model with the help of Example 4 can be viewed in Figure 8.

**Definition 19 .**

Let $\Psi_{𝒟}\in \underset{\text{fpnhs}}{⊎}\left(𝒰\right)$. If $\psi_{𝒟}\left(\hat{g}\right)=\emptyset ,A_{𝒟}\left(\hat{g}\right)=0$ for all $\hat{g}\in 𝔾$, then Ψ_*𝒟*_ is called *𝒟*-empty fpnhs-set, denoted by Ψ_Φ_*𝒟*__. If *𝒟*=∅, then *𝒟*-empty fpnhs-set is called an empty fpnhs-set, denoted by Ψ_Φ_.

**Definition 20 .**

Let $\Psi_{𝒟}\in \underset{\text{fpnhs}}{⊎}\left(𝒰\right)$. If $\psi_{𝒟}\left(\hat{g}\right)=𝒰,A_{𝒟}\left(\hat{g}\right)=1$ for all $\hat{g}\in 𝔾$, then Ψ_*𝒟*_ is called *𝒟*-universal fpnhs-set, denoted by $\Psi_{\tilde{𝒟}}$. If *𝒟*=*𝔾*, then the *𝒟*-universal fpnhs-set is called universal fpnhs-set, denoted by $\Psi_{\tilde{𝔾}}$.

**Example 3 .**

Consider

$𝒰=\left\{{\hat{u}}_{1},{\hat{u}}_{2},{\hat{u}}_{3},{\hat{u}}_{4},{\hat{u}}_{5}\right\}$ and *𝒵*={*𝒵*_1_, *𝒵*_2_, *𝒵*_3_} with ${𝒵}_{1}=\left\{{\hat{z}}_{11},{\hat{z}}_{12}\right\}$, ${𝒵}_{2}=\left\{{\hat{z}}_{21},{\hat{z}}_{22}\right\},{𝒵}_{3}=\left\{{\hat{z}}_{31}\right\}$; then, *𝔾*=*𝒵*_1_ × *𝒵*_2_ × *𝒵*_3_, $𝔾=\left\{\begin{array}{c} \left({\hat{z}}_{11},{\hat{z}}_{21},{\hat{z}}_{31}\right),\left({\hat{z}}_{11},{\hat{z}}_{22},{\hat{z}}_{31}\right),\\ \left({\hat{z}}_{12},{\hat{z}}_{21},{\hat{z}}_{31}\right),\left({\hat{z}}_{12},{\hat{z}}_{22},{\hat{z}}_{31}\right) \end{array}\right\}$, $𝔾=\left\{{\hat{g}}_{1},{\hat{g}}_{2},{\hat{g}}_{3},{\hat{g}}_{4}\right\}$.

Case 1. When ${𝒟}_{1}=\left\{0.2/{\hat{g}}_{2},0.0/{\hat{g}}_{3},1.0/{\hat{g}}_{4}\right\}$, $\psi_{D_{1}}\left({\hat{g}}_{2}\right)=\left\{\begin{array}{c} \left(0.2,0.4,0.6\right)/{\hat{u}}_{2},\\ \left(0.3,0.5,0.7\right)/{\hat{u}}_{4} \end{array}\right\}$, $\psi_{{𝒟}_{1}}\left({\hat{g}}_{3}\right)=\phi$, and $\psi_{{𝒟}_{1}}\left({\hat{g}}_{4}\right)=𝒰$, then
  (19) $$\begin{array}{c} \Psi_{D_{1}}=\left\{\begin{array}{c} \left(\frac{0.2}{{\hat{g}}_{2}},\left\{\begin{array}{c} \frac{\left(0.2,0.4,0.6\right)}{{\hat{u}}_{2}},\\ \frac{\left(0.3,0.5,0.7\right)}{{\hat{u}}_{4}} \end{array}\right\}\right),\\ \left(\frac{0.0}{{\hat{g}}_{3}},\emptyset \right),\left(\frac{1.0}{{\hat{g}}_{4}},U\right) \end{array}\right\}. \end{array}$$
Case 2. When ${𝒟}_{2}=\left\{0.0/{\hat{g}}_{2},0.0/{\hat{g}}_{3}\right\},\psi_{{𝒟}_{2}}\left({\hat{g}}_{2}\right)=\emptyset$, and $\psi_{{𝒟}_{2}}\left({\hat{g}}_{3}\right)=\emptyset$, then Ψ_*𝒟*_2__=Ψ_Φ_*𝒟*_2___.
Case 3. When *𝒟*_3_=∅ for all members of *𝔾*, then Ψ_*𝒟*_3__=Ψ_Φ_.
Case 4. When ${𝒟}_{4}=\left\{1.0/{\hat{g}}_{1},1.0/{\hat{g}}_{2}\right\},\psi_{{𝒟}_{4}}\left({\hat{g}}_{1}\right)=𝒰$, and $\psi_{{𝒟}_{4}}\left({\hat{g}}_{2}\right)=𝒰$, then $\Psi_{{𝒟}_{4}}=\Psi_{\tilde{{𝒟}_{4}}}$.
Case 5. When *𝒟*_5_=*𝒰* for all members of *𝔾*, then $\Psi_{{𝒟}_{5}}=\Psi_{\tilde{𝔾}}$.

**Definition 21 .**

Let Ψ_*𝒟*_1__, $\Psi_{{𝒟}_{2}}\in \underset{\text{fpnhs}}{⊎}\left(𝒰\right)$; then, Ψ_*𝒟*_1__ is an fpnhs-subset of Ψ_*𝒟*_2__, denoted by $\Psi_{{𝒟}_{1}}{\tilde{\subseteq }}_{f}\Psi_{{𝒟}_{2}}$ if$A_{{𝒟}_{1}}\left(\hat{g}\right)\leq A_{{𝒟}_{2}}\left(\hat{g}\right)$ and $\psi_{{𝒟}_{1}}\left(\hat{g}\right)\subseteq_{n}\psi_{{𝒟}_{2}}\left(\hat{g}\right)$ for all $\hat{g}\in 𝔾$.

**Proposition 5 .**

Let $\Psi_{{𝒟}_{1}},\Psi_{{𝒟}_{2}},\Psi_{{𝒟}_{3}}\in \underset{\text{fpnhs}}{⊎}\left(𝒰\right)$; then,

1. $\Psi_{{𝒟}_{1}}{\tilde{\subseteq }}_{f}\Psi_{\tilde{𝔾}}$.
2. $\Psi_{\Phi }{\tilde{\subseteq }}_{f}\Psi_{{𝒟}_{1}}$.
3. $\Psi_{{𝒟}_{1}}{\tilde{\subseteq }}_{f}\Psi_{{𝒟}_{1}}$.
4. If $\Psi_{{𝒟}_{1}}{\tilde{\subseteq }}_{f}\Psi_{{𝒟}_{2}}$ and $\Psi_{{𝒟}_{2}}{\tilde{\subseteq }}_{f}\Psi_{{𝒟}_{3}}\text{, then }\Psi_{{𝒟}_{1}}{\tilde{\subseteq }}_{f}\Psi_{{𝒟}_{3}}$.

**Definition 22 .**

Let $\Psi_{{𝒟}_{1}},\Psi_{{𝒟}_{2}}\in \underset{fpnhs}{⊎}\left(𝒰\right)$; then, Ψ_*𝒟*_1__ and Ψ_*𝒟*_2__ are fpnhs-equal, represented as Ψ_*𝒟*_1__=Ψ_*𝒟*_2__, if and only if $A_{{𝒟}_{1}}\left(\hat{g}\right)=A_{{𝒟}_{2}}\left(\hat{g}\right)$ and $\psi_{{𝒟}_{1}}\left(\hat{g}\right){=}_{n}\psi_{{𝒟}_{2}}\left(\hat{g}\right)$ for all $\hat{g}\in 𝔾$.

**Proposition 6 .**

Let $\Psi_{{𝒟}_{1}},\Psi_{{𝒟}_{2}},\Psi_{{𝒟}_{3}}\in \underset{\text{fpnhs}}{⊎}\left(𝒰\right)$; then:

1. If Ψ_*𝒟*_1__=Ψ_*𝒟*_2__ and Ψ_*𝒟*_2__=Ψ_*𝒟*_3__, then Ψ_*𝒟*_1__=Ψ_*𝒟*_3__.
2. If $\Psi_{{𝒟}_{1}}{\tilde{\subseteq }}_{f}\Psi_{{𝒟}_{2}}$ and $\Psi_{{𝒟}_{2}}{\tilde{\subseteq }}_{f}\Psi_{{𝒟}_{1}}\text{ then }\Psi_{{𝒟}_{1}}=\Psi_{{𝒟}_{2}}$.

**Definition 23 .**

Let $\Psi_{𝒟}\in \underset{\text{fpnhs}}{⊎}\left(𝒰\right)$; then, complement of Ψ_*𝒟*_ (i.e., $\Psi_{𝒟}^{\tilde{c}}$) is an fpnhs-set given as $P_{𝒟}^{\tilde{c}}\left(\hat{g}\right)=1-A_{𝒟}\left(\hat{g}\right)$ and $\psi_{𝒟}^{\tilde{c}}\left(\hat{g}\right)=𝒰\setminus_{n}\psi_{𝒟}\left(\hat{g}\right)$.

**Proposition 7 .**

Let $\Psi_{𝒟}\in \underset{\text{fpnhs}}{⊎}\left(𝒰\right)$; then,

1. ${\left(\Psi_{𝒟}^{\tilde{c}}\right)}^{\tilde{c}}=\Psi_{𝒟}$.
2. $\Psi_{\phi }^{\tilde{c}}=\Psi_{\tilde{𝔾}}$.

**Proof**

The verification of the above parts can be easily obtained from Definition 23.

**Definition 24 .**

Let $\Psi_{{𝒟}_{1}},\Psi_{{𝒟}_{2}}\in \underset{\text{fpnhs}}{⊎}\left(𝒰\right)$; then, union of Ψ_*𝒟*_1__ and Ψ_*𝒟*_2__, denoted by $\Psi_{{𝒟}_{1}}\tilde{\cup }\Psi_{{𝒟}_{2}}$, is a fpnhs-set defined by

- $A_{{𝒟}_{1}\tilde{\cup }{𝒟}_{2}}\left(\hat{g}\right)=\max\left\{A_{{𝒟}_{1}}\left(\hat{g}\right),A_{{𝒟}_{2}}\left(\hat{g}\right)\right\}$.
- $\psi_{{𝒟}_{1}\tilde{\cup }{𝒟}_{2}}\left(\hat{g}\right)=\psi_{{𝒟}_{1}}\left(\hat{g}\right){\tilde{\cup }}_{n}\psi_{{𝒟}_{2}}\left(\hat{g}\right)$, for all $\hat{g}\in 𝔾$.

**Proposition 8 .**

Let $\Psi_{{𝒟}_{1}},\Psi_{{𝒟}_{2}},\Psi_{{𝒟}_{3}}\in \underset{\text{fpnhs}}{⊎}\left(𝒰\right)$; then,

1. $\Psi_{{𝒟}_{1}}\tilde{\cup }\Psi_{{𝒟}_{1}}=\Psi_{{𝒟}_{1}}$,
2. $\Psi_{{𝒟}_{1}}\tilde{\cup }\Psi_{\Phi }=\Psi_{{𝒟}_{1}}$,
3. $\Psi_{{𝒟}_{1}}\tilde{\cup }\Psi_{\tilde{𝔾}}=\Psi_{\tilde{𝔾}}$,
4. $\Psi_{{𝒟}_{1}}\tilde{\cup }\Psi_{{𝒟}_{2}}=\Psi_{{𝒟}_{2}}\tilde{\cup }\Psi_{{𝒟}_{1}}$,
5. $\left(\Psi_{{𝒟}_{1}}\tilde{\cup }\Psi_{{𝒟}_{2}}\right)\tilde{\cup }\Psi_{{𝒟}_{3}}=\Psi_{{𝒟}_{1}}\tilde{\cup }\left(\Psi_{{𝒟}_{2}}\tilde{\cup }\Psi_{{𝒟}_{3}}\right)$.

**Proof**

(1) Taking *𝒟*_1_=*𝒟*_2_ in Definition 24, we have $A_{{𝒟}_{1}\tilde{\cup }{𝒟}_{1}}\left(\hat{g}\right)=\max\left\{A_{{𝒟}_{1}}\left(\hat{g}\right),A_{{𝒟}_{1}}\left(\hat{g}\right)\right\}=A_{{𝒟}_{1}}\left(\hat{g}\right)$ and $\psi_{{𝒟}_{1}\tilde{\cup }{𝒟}_{1}}\left(\hat{g}\right)=\psi_{{𝒟}_{1}}\left(\hat{g}\right){\tilde{\cup }}_{n}\psi_{{𝒟}_{1}}\left(\hat{g}\right)$, for all $\hat{g}\in 𝔾$, which implies $\psi_{{𝒟}_{1}\tilde{\cup }{𝒟}_{1}}\left(\hat{g}\right)=\psi_{{𝒟}_{1}}\left(\hat{g}\right)$.
Parts (2)–(4) can be proved in a similar manner.
(5) Since
  (20) $$\begin{array}{c} A_{D_{1}\tilde{\cup }\left(D_{2}\tilde{\cup }D_{3}\right)}\left(\hat{g}\right)=\max\left\{A_{D_{1}}\left(\hat{g}\right),A_{D_{2}\tilde{\cup }D_{3}}\left(\hat{g}\right)\right\}\\ =\max\left\{A_{D_{1}}\left(\hat{g}\right),\max\left\{A_{D_{2}}\left(\hat{g}\right),A_{D_{3}}\left(\hat{g}\right)\right\}\right\}\\ =\max\left\{A_{D_{1}}\left(\hat{g}\right),\left\{A_{D_{2}}\left(\hat{g}\right),A_{D_{3}}\left(\hat{g}\right)\right\}\right\}\\ =\max\left\{\left\{A_{D_{1}}\left(\hat{g}\right),A_{D_{2}}\left(\hat{g}\right)\right\},A_{D_{3}}\left(\hat{g}\right)\right\}\\ =\max\left\{\max\left\{A_{D_{1}}\left(\hat{g}\right),A_{D_{2}}\left(\hat{g}\right)\right\},A_{D_{3}}\left(\hat{g}\right)\right\}\\ =\max\left\{A_{D_{1}\tilde{\cup }D_{2}}\left(\hat{g}\right),A_{D_{3}}\left(\hat{g}\right)\right\}\\ =A_{\left(D_{1}\tilde{\cup }D_{2}\right)\tilde{\cup }D_{3}}\left(\hat{g}\right), \end{array}$$
and
  (21) $$\begin{array}{c} \psi_{D_{1}{\tilde{\cup }}_{n}\left(D_{2}{\tilde{\cup }}_{n}D_{3}\right)}\left(\hat{g}\right)=\psi_{D_{1}}\left(\hat{g}\right){\tilde{\cup }}_{n}\psi_{D_{2}{\tilde{\cup }}_{n}D_{3}}\left(\hat{g}\right)\\ =\psi_{D_{1}}\left(\hat{g}\right){\tilde{\cup }}_{n}\left(\psi_{D_{2}}\left(\hat{g}\right){\tilde{\cup }}_{n}\psi_{D_{3}}\left(\hat{g}\right)\right)\\ =\left(\psi_{D_{1}}\left(\hat{g}\right){\tilde{\cup }}_{n}\psi_{D_{2}}\left(\hat{g}\right)\right){\tilde{\cup }}_{n}\psi_{D_{3}}\left(\hat{g}\right)\\ =\psi_{D_{1}{\tilde{\cup }}_{n}D_{2}}\left(\hat{g}\right){\tilde{\cup }}_{n}\psi_{D_{3}}\left(\hat{g}\right)\\ =\psi_{\left(D_{1}{\tilde{\cup }}_{n}D_{2}\right){\tilde{\cup }}_{n}D_{3}}\left(\hat{g}\right). \end{array}$$

**Definition 25 .**

Let $\Psi_{{𝒟}_{1}},\Psi_{{𝒟}_{2}}\in \underset{\text{fpnhs}}{⊎}\left(𝒰\right)$; then, intersection of Ψ_*𝒟*_1__ and Ψ_*𝒟*_2__, denoted by $\Psi_{{𝒟}_{1}}\tilde{\cap }\Psi_{{𝒟}_{2}}$, is a fpnhs-set defined by

- $A_{{𝒟}_{1}\tilde{\cap }{𝒟}_{2}}\left(\hat{g}\right)=\min\left\{A_{{𝒟}_{1}}\left(\hat{g}\right),A_{{𝒟}_{2}}\left(\hat{g}\right)\right\}$.
- $\psi_{{𝒟}_{1}\tilde{\cap }{𝒟}_{2}}\left(\hat{g}\right)=\psi_{{𝒟}_{1}}\left(\hat{g}\right){\tilde{\cap }}_{n}\psi_{{𝒟}_{2}}\left(\hat{g}\right)$, for all $\hat{g}\in 𝔾$.

**Proposition 9 .**

Let $\Psi_{{𝒟}_{1}},\Psi_{{𝒟}_{2}},\Psi_{{𝒟}_{3}}\in \underset{\text{fpnhs}}{⊎}\left(𝒰\right)$; then

1. $\Psi_{{𝒟}_{1}}\tilde{\cap }\Psi_{{𝒟}_{1}}=\Psi_{{𝒟}_{1}}$.
2. $\Psi_{{𝒟}_{1}}\tilde{\cap }\Psi_{\Phi }=\Psi_{\Phi }$.
3. $\Psi_{{𝒟}_{1}}\tilde{\cap }\Psi_{\tilde{𝔾}}=\Psi_{\tilde{{𝒟}_{1}}}$.
4. $\Psi_{{𝒟}_{1}}\tilde{\cap }\Psi_{{𝒟}_{2}}=\Psi_{{𝒟}_{2}}\tilde{\cap }\Psi_{{𝒟}_{1}}$.
5. $\left(\Psi_{{𝒟}_{1}}\tilde{\cap }\Psi_{{𝒟}_{2}}\right)\tilde{\cap }\Psi_{\Psi_{{𝒟}_{3}}}=\Psi_{{𝒟}_{1}}\tilde{\cap }\left(\Psi_{{𝒟}_{2}}\tilde{\cap }\Psi_{\Psi_{{𝒟}_{3}}}\right)$.

**Proof**

(1) Taking *𝒟*_1_=*𝒟*_2_ in Definition 25, we have $A_{{𝒟}_{1}\tilde{\cap }{𝒟}_{1}}\left(\hat{g}\right)=\min\left\{A_{{𝒟}_{1}}\left(\hat{g}\right),A_{{𝒟}_{1}}\left(\hat{g}\right)\right\}=A_{{𝒟}_{1}}\left(\hat{g}\right)$ and $\psi_{{𝒟}_{1}\tilde{\cap }{𝒟}_{1}}\left(\hat{g}\right)=\psi_{{𝒟}_{1}}\left(\hat{g}\right){\tilde{\cap }}_{n}\psi_{{𝒟}_{1}}\left(\hat{g}\right)$, for all $\hat{g}\in 𝔾$, which implies $\psi_{{𝒟}_{1}\tilde{\cap }{𝒟}_{1}}\left(\hat{g}\right)=\psi_{{𝒟}_{1}}\left(\hat{g}\right)$.
Parts (2)–(4) can also be shown in a similar manner.
(5) Since
  (22) $$\begin{array}{c} A_{D_{1}\tilde{\cap }\left(D_{2}\tilde{\cap }D_{3}\right)}\left(\hat{g}\right)=\min\left\{A_{D_{1}}\left(\hat{g}\right),A_{D_{2}\tilde{\cap }D_{3}}\left(\hat{g}\right)\right\}\\ =\min\left\{A_{D_{1}}\left(\hat{g}\right),\min\left\{A_{D_{2}}\left(\hat{g}\right),A_{D_{3}}\left(\hat{g}\right)\right\}\right\}\\ =\min\left\{A_{D_{1}}\left(\hat{g}\right),\left\{A_{D_{2}}\left(\hat{g}\right),A_{D_{3}}\left(\hat{g}\right)\right\}\right\}\\ =\min\left\{\left\{A_{D_{1}}\left(\hat{g}\right),A_{D_{2}}\left(\hat{g}\right)\right\},A_{D_{3}}\left(\hat{g}\right)\right\}\\ =\min\left\{\min\left\{A_{D_{1}}\left(\hat{g}\right),A_{D_{2}}\left(\hat{g}\right)\right\},A_{D_{3}}\left(\hat{g}\right)\right\}\\ =\min\left\{A_{D_{1}\tilde{\cap }D_{2}}\left(\hat{g}\right),A_{D_{3}}\left(\hat{g}\right)\right\}\\ =A_{\left(D_{1}\tilde{\cap }D_{2}\right)\tilde{\cap }D_{3}}\left(\hat{g}\right), \end{array}$$
and
  (23) $$\begin{array}{c} \psi_{D_{1}{\tilde{\cap }}_{n}\left(D_{2}{\tilde{\cap }}_{n}D_{3}\right)}\left(\hat{g}\right)=\psi_{D_{1}}\left(\hat{g}\right){\tilde{\cap }}_{n}\psi_{D_{2}{\tilde{\cap }}_{n}D_{3}}\left(\hat{g}\right)\\ =\psi_{D_{1}}\left(\hat{g}\right){\tilde{\cap }}_{n}\left(\psi_{D_{2}}\left(\hat{g}\right){\tilde{\cap }}_{n}\psi_{D_{3}}\left(\hat{g}\right)\right)\\ =\left(\psi_{D_{1}}\left(\hat{g}\right){\tilde{\cap }}_{n}\psi_{D_{2}}\left(\hat{g}\right)\right){\tilde{\cap }}_{n}\psi_{D_{3}}\left(\hat{g}\right)\\ =\psi_{D_{1}{\tilde{\cap }}_{n}D_{2}}\left(\hat{g}\right){\tilde{\cap }}_{n}\psi_{D_{3}}\left(\hat{g}\right)\\ =\psi_{\left(D_{1}{\tilde{\cap }}_{n}D_{2}\right){\tilde{\cap }}_{n}D_{3}}\left(\hat{g}\right). \end{array}$$

Note. It is pertinent to mention here that Propositions 5, 6, 8, and 9 are also valid for elements of $\underset{\text{fpifhs}}{⊎}\left(𝒰\right)$.

**Remark 2 .**

Let $\Psi_{𝒟}\in \underset{\text{fpnhs}}{⊎}\left(𝒰\right)$. If $\Psi_{𝒟}\neq \Psi_{\tilde{𝔾}}$, then $\Psi_{𝒟}\tilde{\cup }\Psi_{𝒟}^{\tilde{c}}\neq \Psi_{\tilde{𝔾}}$ and $\Psi_{𝒟}\tilde{\cap }\Psi_{𝒟}^{\tilde{c}}\neq \Psi_{\Phi }$.

**Proposition 10 .**

Let $\Psi_{{𝒟}_{1}},\Psi_{{𝒟}_{2}}\in \underset{\text{fpnhs}}{⊎}\left(𝒰\right)$; then, the following De Morgan laws are valid:

1. ${\left(\Psi_{{𝒟}_{1}}\tilde{\cup }\Psi_{{𝒟}_{2}}\right)}^{\tilde{c}}=\Psi_{{𝒟}_{1}}^{\tilde{c}}\tilde{\cap }\Psi_{{𝒟}_{2}}^{\tilde{c}}$.
2. ${\left(\Psi_{{𝒟}_{1}}\tilde{\cap }\Psi_{{𝒟}_{2}}\right)}^{\tilde{c}}=\Psi_{{𝒟}_{1}}^{\tilde{c}}\tilde{\cup }\Psi_{{𝒟}_{2}}^{\tilde{c}}$.

**Proof**

For all $\hat{g}\in 𝔾$:

(1) Since
  (24) $$\begin{array}{c} {\left(A_{D_{1}\tilde{\cup }D_{2}}\right)}^{\tilde{c}}\left(\hat{g}\right)=1-A_{D_{1}\tilde{\cup }D_{2}}\left(\hat{g}\right)\\ =1-\max\left\{A_{D_{1}}\left(\hat{g}\right),A_{D_{2}}\left(\hat{g}\right)\right\}\\ =\min\left\{1-A_{D_{1}}\left(\hat{g}\right),1-A_{D_{2}}\left(\hat{g}\right)\right\}\\ =\min\left\{P_{D_{1}}^{\tilde{c}}\left(\hat{g}\right),P_{D_{2}}^{\tilde{c}}\left(\hat{g}\right)\right\}\\ =P_{D_{1}\tilde{\cap }D_{2}}^{\tilde{c}}\left(\hat{g}\right), \end{array}$$
and
  (25) $$\begin{array}{c} {\left(\psi_{D_{1}{\tilde{\cup }}_{n}D_{2}}\right)}^{\tilde{c}}\left(\hat{g}\right)=U\setminus_{n}\psi_{D_{1}{\tilde{\cup }}_{n}D_{2}}\left(\hat{g}\right)\\ =U\setminus_{n}\left(\psi_{D_{1}}\left(\hat{g}\right){\tilde{\cup }}_{n}\psi_{D_{2}}\left(\hat{g}\right)\right)\\ =\left(U\setminus_{n}\psi_{D_{1}}\left(\hat{g}\right)\right){\tilde{\cap }}_{n}\left(U\setminus_{n}\psi_{D_{2}}\left(\hat{g}\right)\right)\\ =\psi_{D_{1}}^{\tilde{c}}\left(\hat{g}\right)\tilde{\cap_{n}}\psi_{D_{2}}^{\tilde{c}}\left(\hat{g}\right)\\ =\psi_{D_{1}{\tilde{\cap }}_{n}D_{2}}^{\tilde{c}}\left(\hat{g}\right). \end{array}$$

By adopting the same method, the second part may easily be proved.

**Proposition 11 .**

Let $\Psi_{{𝒟}_{1}},\Psi_{{𝒟}_{2}},\Psi_{{𝒟}_{3}}\in \underset{\text{fpnhs}}{⊎}\left(𝒰\right)$; then:

1. $\Psi_{{𝒟}_{1}}\tilde{\cup }\left(\Psi_{{𝒟}_{2}}\tilde{\cap }\Psi_{{𝒟}_{3}}\right)=\left(\Psi_{{𝒟}_{1}}\tilde{\cup }\Psi_{{𝒟}_{2}}\right)\tilde{\cap }\left(\Psi_{{𝒟}_{1}}\tilde{\cup }\Psi_{{𝒟}_{3}}\right)$.
2. $\Psi_{{𝒟}_{1}}\tilde{\cap }\left(\Psi_{{𝒟}_{2}}\tilde{\cup }\Psi_{{𝒟}_{3}}\right)=\left(\Psi_{{𝒟}_{1}}\tilde{\cap }\Psi_{{𝒟}_{2}}\right)\tilde{\cup }\left(\Psi_{{𝒟}_{1}}\tilde{\cap }\Psi_{{𝒟}_{3}}\right)$.

**Proof**

For all $\hat{g}\in 𝔾$:

(1) Since
  (26) $$\begin{array}{c} A_{D_{1}\tilde{\cup }\left(D_{2}\tilde{\cap }D_{3}\right)}\left(\hat{g}\right)=\max\left\{A_{D_{1}}\left(\hat{g}\right),A_{D_{2}\tilde{\cap }D_{3}}\left(\hat{g}\right)\right\}\\ =\max\left\{A_{D_{1}}\left(\hat{g}\right),\min\left\{A_{D_{2}}\left(\hat{g}\right),A_{D_{3}}\left(\hat{g}\right)\right\}\right\}\\ =\min\left\{\max\left\{A_{D_{1}}\left(\hat{g}\right),A_{D_{2}}\left(\hat{g}\right)\right\},\max\left\{A_{D_{1}}\left(\hat{g}\right),A_{D_{3}}\left(\hat{g}\right)\right\}\right\}\\ =\min\left\{A_{D_{1}\tilde{\cup }D_{2}}\left(\hat{g}\right),A_{D_{1}\tilde{\cup }D_{3}}\left(\hat{g}\right)\right\}\\ =A_{\left(D_{1}\tilde{\cup }D_{2}\right)\tilde{\cap }\left(D_{1}\tilde{\cup }D_{3}\right)}\left(\hat{g}\right), \end{array}$$
and
  (27) $$\begin{array}{c} \psi_{D_{1}{\tilde{\cup }}_{n}\left(D_{2}{\tilde{\cap }}_{n}D_{3}\right)}\left(\hat{g}\right)=\psi_{D_{1}}\left(\hat{g}\right){\tilde{\cup }}_{n}\psi_{D_{2}{\tilde{\cap }}_{n}D_{3}}\left(\hat{g}\right)\\ =\psi_{D_{1}}\left(\hat{g}\right){\tilde{\cup }}_{n}\left(\psi_{D_{2}}\left(\hat{g}\right){\tilde{\cap }}_{n}\psi_{D_{3}}\left(\hat{g}\right)\right)\\ =\left(\psi_{D_{1}}\left(\hat{g}\right){\tilde{\cup }}_{n}\psi_{D_{2}}\left(\hat{g}\right)\right){\tilde{\cap }}_{n}\left(\psi_{D_{1}}\left(\hat{g}\right){\tilde{\cup }}_{n}\psi_{D_{3}}\left(\hat{g}\right)\right)\\ =\psi_{D_{1}{\tilde{\cup }}_{n}D_{2}}\left(\hat{g}\right){\tilde{\cap }}_{n}\psi_{D_{1}{\tilde{\cup }}_{n}D_{3}}\left(\hat{g}\right)\\ =\psi_{\left(D_{1}{\tilde{\cup }}_{n}D_{2}\right){\tilde{\cap }}_{n}\left(D_{1}{\tilde{\cup }}_{n}D_{3}\right)}\left(\hat{g}\right). \end{array}$$

The same method may be used to prove the second part.

**Definition 26 .**

Let $\Psi_{{𝒟}_{1}},\Psi_{{𝒟}_{2}}\in \underset{\text{fpnhs}}{⊎}\left(𝒰\right)$; then, OR-operation of Ψ_*𝒟*_1__ and Ψ_*𝒟*_2__, denoted by $\Psi_{{𝒟}_{1}}\tilde{⊕}\Psi_{{𝒟}_{2}}$, is a fpnhs-set defined by

- $A_{{𝒟}_{1}\tilde{⊕}{𝒟}_{2}}\left({\hat{g}}_{1},{\hat{g}}_{2}\right)=\max\left\{A_{{𝒟}_{1}}\left({\hat{g}}_{1}\right),A_{{𝒟}_{2}}\left({\hat{g}}_{2}\right)\right\}$.
- $\psi_{{𝒟}_{1}\tilde{⊕}{𝒟}_{2}}\left({\hat{g}}_{1},{\hat{g}}_{2}\right)=\psi_{{𝒟}_{1}}\left({\hat{g}}_{1}\right){\tilde{\cup }}_{n}\psi_{{𝒟}_{2}}\left({\hat{g}}_{2}\right)$, for all $\left({\hat{g}}_{1},{\hat{g}}_{2}\right)\in {𝒟}_{1}\times {𝒟}_{2}$.

**Definition 27 .**

Let $\Psi_{{𝒟}_{1}},\Psi_{{𝒟}_{2}}\in \underset{\text{fpnhs}}{⊎}\left(𝒰\right)$; then, AND-operation of Ψ_*𝒟*_1__ and Ψ_*𝒟*_2__, denoted by $\Psi_{{𝒟}_{1}}\tilde{⊗}\Psi_{{𝒟}_{2}}$, is a fpnhs-set defined by

- $A_{{𝒟}_{1}\tilde{⊗}{𝒟}_{2}}\left({\hat{g}}_{1},{\hat{g}}_{2}\right)=\min\left\{A_{{𝒟}_{1}}\left({\hat{g}}_{1}\right),A_{{𝒟}_{2}}\left({\hat{g}}_{2}\right)\right\}$.
- $\psi_{{𝒟}_{1}\tilde{⊗}{𝒟}_{2}}\left({\hat{g}}_{1},{\hat{g}}_{2}\right)=\psi_{{𝒟}_{1}}\left({\hat{g}}_{1}\right){\tilde{\cap }}_{n}\psi_{{𝒟}_{2}}\left({\hat{g}}_{2}\right)$, for all $\left({\hat{g}}_{1},{\hat{g}}_{2}\right)\in {𝒟}_{1}\times {𝒟}_{2}$.

**Proposition 12 .**

Let $\Psi_{{𝒟}_{1}},\Psi_{{𝒟}_{2}},\Psi_{{𝒟}_{3}}\in \underset{\text{fpnhs}}{⊎}\left(𝒰\right)$; then,

1. $\Psi_{{𝒟}_{1}}\tilde{⊗}\Psi_{\Phi }=\Psi_{\Phi }$.
2. $\left(\Psi_{{𝒟}_{1}}\tilde{⊗}\Psi_{{𝒟}_{2}}\right)\tilde{⊗}\Psi_{{𝒟}_{3}}=\Psi_{{𝒟}_{1}}\tilde{⊗}\left(\Psi_{{𝒟}_{2}}\tilde{⊗}\Psi_{{𝒟}_{3}}\right)$.
3. $\left(\Psi_{{𝒟}_{1}}\tilde{⊕}\Psi_{{𝒟}_{2}}\right)\tilde{⊕}\Psi_{{𝒟}_{3}}=\Psi_{{𝒟}_{1}}\tilde{⊕}\left(\Psi_{{𝒟}_{2}}\tilde{⊕}\Psi_{{𝒟}_{3}}\right)$.

**Proof**

The above parts can easily be verified with the help of Definitions 26 and 27.

### 4.1. Fuzzy Decision Set of fpnhs-Set

This section presents the conceptualization of fuzzy decision set for fpnhs-set to solve decision-making problems via proposed algorithm and example.

**Definition 28 .**

Let $\Psi_{𝒟}\in \underset{\text{fpnhs}}{⊎}\left(𝒰\right)$; then, a fuzzy decision set of Ψ_*𝒟*_ (i.e., Ψ_*𝒟*_^*D*^) is represented as

(28) $$\begin{array}{c} \Psi_{D}^{D}=\left\{\frac{\langle T_{D}^{D}\left(\hat{u}\right)\rangle }{\hat{u}:\hat{u}\in U}\right\}, \end{array}$$

where *𝒯*_*𝒟*_^*D*^ : *𝒰*⟶*𝕀* and

(29) $$\begin{array}{c} T_{D}^{D}\left(\hat{u}\right)=\frac{1}{\left|U\right|}\sum_{v\in S\left(D\right)}T_{D}\left(v\right)\Gamma_{\psi_{D}\left(v\right)}\left(\hat{u}\right), \end{array}$$

where |•| denotes set cardinality with

(30) $$\begin{array}{c} \Gamma_{\psi_{D}\left(v\right)}\left(\hat{u}\right)=\left\{\begin{array}{cc} \left|T_{\psi_{D}}\left(\hat{u}\right)+I_{\psi_{D}}\left(\hat{u}\right)-F_{\psi_{D}}\left(\hat{u}\right)\right|, & u\in \Gamma_{\psi_{D}}\left(v\right),\\ 0, & \hat{u}\notin \Gamma_{\psi_{D}}\left(v\right). \end{array}\right. \end{array}$$

Whenever Ψ_*𝒟*_^*D*^ has been established, it might be relevant to select the suitable single substitute from the available options. Therefore, the following algorithm may lead to the final decisive selection.

**Figure 9:**
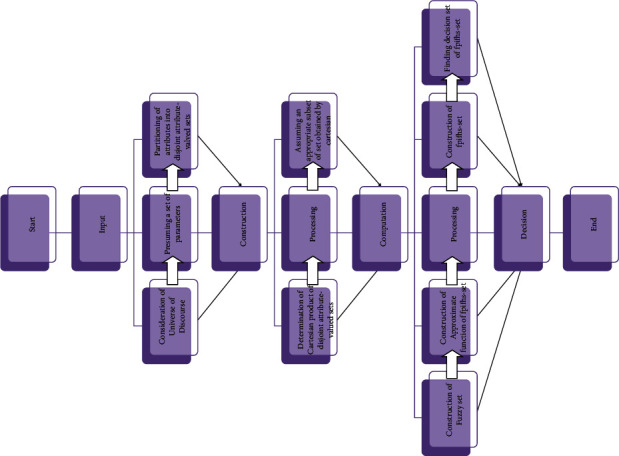
Decision-making algorithm for fpnhs-set.

Figure 9 presents the flowchart of Algorithm 2.

**Example 4 .**

**Figure 10:**
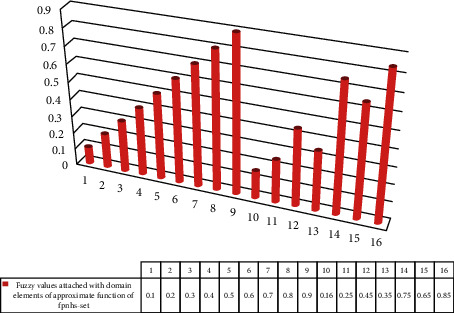
Graphical representation of Table 5.

**Figure 11:**
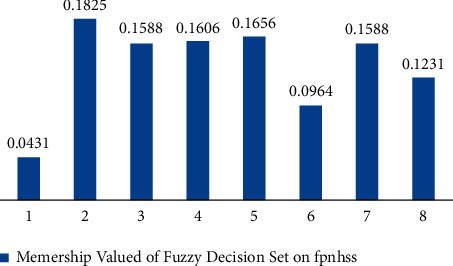
Fuzzy decision system on FPNHS-set.

Input and Construction Stages (1–5):
Considering the data given in Example 2, we have
Computation Stage (6–9):
(6) The graphical depiction of Table 5 can be seen in Figure 10.
From Table 5, we can construct *𝒟* as
  (31) $$\begin{array}{c} D=\left\{\begin{array}{c} \frac{0.1}{p^{1}},\frac{0.2}{p^{2}},\frac{0.3}{p^{3}},\frac{0.4}{p^{4}},\\ \frac{0.5}{p^{5}},\frac{0.6}{p^{6}},\frac{0.7}{p^{7}},\frac{0.8}{p^{8}},\\ \frac{0.9}{p^{9}},\frac{0.16}{p^{10}}\frac{0.25}{p^{11}},\frac{0.45}{p^{12}},\\ \frac{0.35}{p^{13}},\frac{0.75}{p^{14}},\frac{0.65}{p^{15}},\frac{0.85}{p^{16}} \end{array}\right\}. \end{array}$$
(7) Table 6 presents *ψ*_*𝒟*_(*p*^*i*^) corresponding to each element of *𝔾*.
(8) Ψ_*𝒟*_ can be constructed with the help of step 6 and step 7 in the same way as step 8 of Section 3.
(9) From Table 7, we can construct Ψ_*𝒟*_^*D*^ as
  (32) $$\begin{array}{c} \Psi_{D}^{D}=\left\{\begin{array}{c} \frac{0.0431}{{ℍ}^{1}},\frac{0.1825}{{ℍ}^{2}},\frac{0.1588}{{ℍ}^{3}},\\ \frac{0.1606}{{ℍ}^{4}},\frac{0.1656}{{ℍ}^{5}},\frac{0.0964}{{ℍ}^{6}},\\ \frac{0.1588}{{ℍ}^{7}},\frac{0.1231}{{ℍ}^{8}} \end{array}\right\}. \end{array}$$
Please see Figure 11 for its pictorial depiction.
Output Stage:
(10) Since maximum of *𝒯*_*𝒟*_^*D*^(ℍ^*i*^) is 0.1825, the hand sanitizer ℍ^2^ is selected.

## 5. Comparison Analysis

**Figure 12:**
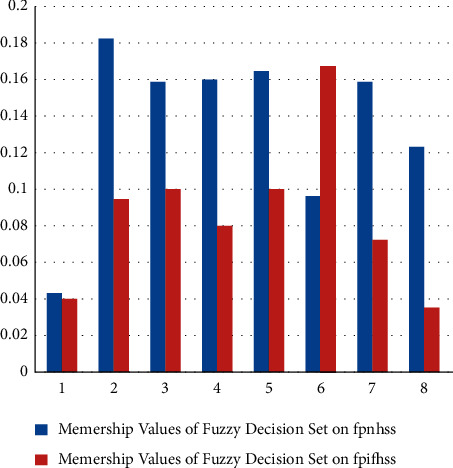
Comparison of membership values of fuzzy decision sets on fpifhs-set and fpnhs-set.

There are many cases where consideration of attributes only is not sufficient; all available distinct attributes are further partitioned into their respective disjoint attributive sets. Decision-making techniques based on existing soft set-like models are inadequate for such cases. Therefore, our proposed models not only emphasize the due status of such partitioning of attributes, but also enable the decision-makers to deal with daily-life problems with great ease. Tables 8 and 9 present a clear comparison of our proposed models with some existing models under the evaluating indicators MD (membership degree), NMD (nonmembership degree), ID (indeterminacy degree), SAAF (single-argument approximate function), and MAAF (multiargument approximate function).  Figure 12 depicts the comparison of reduced fuzzy values obtained from our proposed structures.

## 6. Discussion

Decision-makers consistently face a type of uncertainty and the decision taken by overlooking uncertainty might have some degree of tendency. Indeterminacy and uncertainty are both interconnected and interrelated. The proposed work has elaborated (for example, see Figure 12) how the results are affected by the omission or consideration of some uncertain components while dealing with the real-world problems under any decisive system. Two models fpifhs-set and fpnhs-set have been proposed and characterized. The latter one is very useful, flexible, and reliable in dealing with problems having uncertain information and data. The following discussion shows the generalization of fpnhs-set as it fulfills all the characteristics, features, and properties of many existing soft set-like models. In Definition 18:

- If *ℕ*(*𝒰*) is replaced with *𝕀𝔽*(*𝒰*), it becomes fpifhs-set.
- If *ℕ*(*𝒰*) is replaced with *𝔽*(*𝒰*), it behaves as fpfhs-set.
- If *ℕ*(*𝒰*) is replaced with ℙ(*𝒰*), it converts to fphs-set.
- If *𝔾*=*𝒮*_1_ × *𝒮*_2_ × *𝒮*_3_ × ⋯·×*𝒮*_*n*_ is omitted and only set of parameters is considered, then it is called fpns-set.
- If only set of parameters is considered after omitting *𝔾*=*𝒮*_1_ × *𝒮*_2_ × *𝒮*_3_ × ⋯·×*𝒮*_*n*_ and replacing *ℕ*(*𝒰*) with *𝕀𝔽*(*𝒰*), then it is called fpifs-set.
- If only set of parameters is considered after omitting *𝔾*=*𝒮*_1_ × *𝒮*_2_ × *𝒮*_3_ × ⋯·×*𝒮*_*n*_ and replacing *ℕ*(*𝒰*) with *𝔽*(*𝒰*), then it is known as fpfs-set.
- If $A_{𝒟}\left(\hat{g}\right)/\hat{g}$ is replaced with $\hat{g}$, then it is called *nhs*-set.
- If $A_{𝒟}\left(\hat{g}\right)/\hat{g}$ is replaced with $\hat{g}$, and *ℕ*(*𝒰*) is replaced with *𝕀𝔽*(*𝒰*), then it is called ifhs-set.
- If $A_{𝒟}\left(\hat{g}\right)/\hat{g}$ is replaced with $\hat{g}$, and *ℕ*(*𝒰*) is replaced with *𝔽*(*𝒰*), then it is called fhs-set.

**Figure 13:**
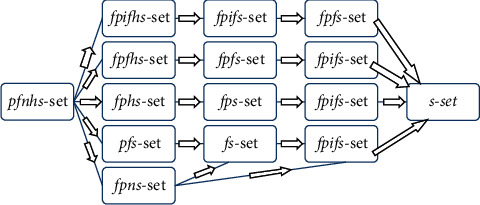
Generalization of the proposed structure.

Similarly, fpnhs-set transforms to *s*-set by ignoring all uncertain components and considering only set of attributes. From Figure 13, it is obvious that the proposed structure is the most generalized structure satisfying all the main characteristics of existing relevant structures.

### 6.1. Advantages of Proposed Research

Now, some advantages of the proposed structure (i.e., fpnhs-set) are presented below:

- The proposed technique took the importance of the concept of parameterization along with the *nhs*-set for dealing with decision-making problems. The considered parameterized fuzzy degree mirrors the possibility of the existence of the level of acknowledgment and excusal; along these lines, this association has marvelous prospective in the legitimate representation inside the space of computational incursions.
- Due to focus on deep observation of parameters and respective attribute-valued sets, the proposed model may assist the decision-makers to have flexible and reliable decisions through decision-making.
- It validates all the qualities and properties of the predefined models so it is not irrational to consider it as the generalized version of these models.

The advantage of the proposed structure can easily be judged from Tables 8 and 9. The comparison is evaluated on the basis of two different aspects:

1. Main features discussed in the study (see Table 8).
2. Features like MD (membership degree), NMD (nonmembership degree), ID (indeterminacy degree), SAAF (single-argument approximate function), and MAAF (multiargument approximate function) (see Table 9).

## 7. Conclusion

The key features of this work can be summarized as follows:

(1) The fundamental theory of fpifhs-set is developed, and its decision-support system is constructed. A real-world problem (an optimum selection of hand sanitizer) is discussed with the support of the proposed algorithm and decision system of fpifhs-set.
(2) The rudiments of fpnhs-set are characterized, and its decision-making based system is developed. A real-life problem (an optimum selection of hand sanitizer) is studied with the help of proposed algorithm and decision system of fpnhs-set.
(3) Both proposed models, fpifhs-set and fpnhs-set, are compared professionally via inter-cum-intra-strategy with some existing relevant models in view of important evaluating features.
(4) The particular cases of the proposed models fpifhs-set and fpnhs-set are discussed with the generalization of these structures.
(5) Although the proposed structure is generalized and reliable for decision-making scenarios, it is flexible due to some limitations; for example, it is inadequate for the situation when the inclusion of nonmembership grade and indeterminate grade in the domain of multiargument approximate function is mandatory. Therefore, future work may include the following:
  - The extension of this study to develop intuitionistic fuzzy parameterized neutrosophic hypersoft set and neutrosophic parameterized neutrosophic hypersoft set to tackle the situations having mandatory inclusion of nonmembership grade and indeterminate grade in the domain of multiargument approximate function.
  - The application of this study to discuss real-world problems using multicriteria decision-making approaches like TOPSIS, PROMETHEE, and MULTIMORA.
  - The application of this study to discuss real-world problems (e.g., pattern recognition) using similarity measures, like cosine similarity, cotangent similarity, and Dice similarity, and entropy measures. [51].

## Figures and Tables

**Algorithm 1:**
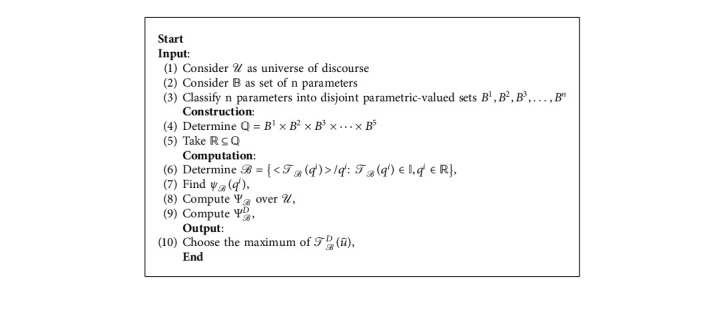
Optimal selection of hand sanitizer by using fpifhs-set.

**Algorithm 2:**
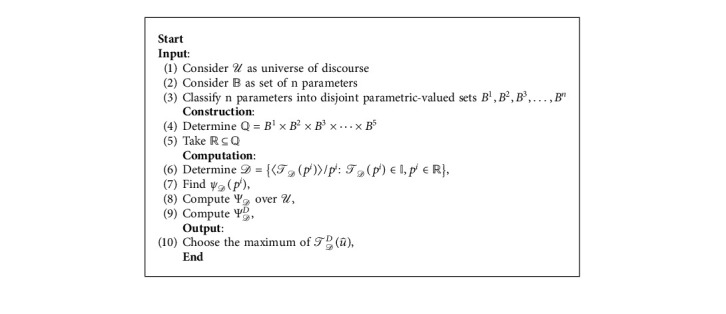
Optimal selection of hand sanitizer by using fpnhs-set.

**Table 1:** Literature review on the parameterization of fuzzy set-like models under soft set.

Authors	Structure	Domain parameterization	Range setting
Adam and Hassan [40]	Multi *Q*-fuzzy parameterized soft set	Multi *Q*-fuzzy set	Soft set
Alkhazaleh et al. [41]	Fuzzy parameterized interval-valued fuzzy soft set	Fuzzy set	Soft set
Aydın and Enginoğlu [42]	Interval-valued intuitionistic fuzzy parameterized interval-valued intuitionistic fuzzy soft set	Interval-valued intuitionistic fuzzy set	Interval-valued intuitionistic fuzzy soft set
Broumi et al. [21]	Neutrosophic parameterized soft set (*nps*-set)	Neutrosophic set	Soft set
Çağman et al. [43]	Fuzzy parameterized fuzzy soft set (fpfs-set)	Fuzzy set	Fuzzy soft set
Deli and Çağman [44]	Intuitionistic fuzzy parameterized soft set (ifps-set)	Intuitionistic fuzzy set	Soft set
Hassan and Al-Qudah [45]	Fuzzy parameterized complex multi-fuzzy soft set (fpcmfs-set)	Fuzzy set	Complex multi-fuzzy soft set
Hazaymeh et al. [46]	Fuzzy parameterized fuzzy soft expert set (fpfse-set)	Fuzzy set	Fuzzy soft expert set
Joshi et al. [47]	Intuitionistic fuzzy parameterized fuzzy soft set (ifpfs-set)	Intuitionistic fuzzy set	Fuzzy soft set
Karaaslan [48]	Intuitionistic fuzzy parameterized intuitionistic fuzzy soft set (ifpifs-set)	Intuitionistic fuzzy set	Intuitionistic fuzzy soft set
Riaz and Hashmi [49]	Fuzzy parameterized fuzzy soft set (fpfs-set)	Fuzzy set	Fuzzy soft set
Zhu and Zhan [50]	Fuzzy parameterized fuzzy soft set (fpfs-set)	Fuzzy set	Fuzzy soft set

**Table 2:** Degrees of membership *𝒯*_ℬ_(*q*^*i*^).

*𝒯*_ℬ_(*q*^*i*^)	Degree	*𝒯*_ℬ_(*q*^*i*^)	Degree	*𝒯*_ℬ_(*q*^*i*^)	Degree	*𝒯*_ℬ_(*q*^*i*^)	Degree
*𝒯*_ℬ_(*q*^1^)	0.1	*𝒯*_ℬ_(*q*^5^)	0.5	*𝒯*_ℬ_(*q*^9^)	0.9	*𝒯*_ℬ_(*q*^13^)	0.35
*𝒯*_ℬ_(*q*^2^)	0.2	*𝒯*_ℬ_(*q*^6^)	0.6	*𝒯*_ℬ_(*q*^10^)	0.16	*𝒯*_ℬ_(*q*^14^)	0.75
*𝒯*_ℬ_(*q*^3^)	0.3	*𝒯*_ℬ_(*q*^7^)	0.7	*𝒯*_ℬ_(*q*^11^)	0.25	*𝒯*_ℬ_(*q*^15^)	0.65
*𝒯*_ℬ_(*q*^4^)	0.4	*𝒯*_ℬ_(*q*^8^)	0.8	*𝒯*_ℬ_(*q*^12^)	0.45	*𝒯*_ℬ_(*q*^16^)	0.85

**Table 3:** Approximate functions *ψ*_ℬ_(*q*^*i*^).

*q* ^*i*^	*ψ*_ℬ_(*q*^*i*^)	*q* ^*i*^	*ψ*_ℬ_(*q*^*i*^)
*q* ^1^	$\left\{\begin{array}{c} \left(0.2,0.1\right)/{ℍ}^{1},\left(0.3,0.2\right)/{ℍ}^{2} \end{array}\right\}$	*q* ^9^	$\left\{\begin{array}{c} \left(0.4,0.3\right)/{ℍ}^{2},\left(0.6,0.4\right)/{ℍ}^{7},\left(0.5,0.4\right)/{ℍ}^{8} \end{array}\right\}$
*q* ^2^	$\left\{\begin{array}{c} \left(0.1,0.2\right)/{ℍ}^{1},\left(0.5,0.4\right)/{ℍ}^{2},\left(0.1,0.4\right)/{ℍ}^{3} \end{array}\right\}$	*q* ^10^	$\left\{\begin{array}{c} \left(0.2,0.1\right)/{ℍ}^{6},\left(0.6,0.4\right)/{ℍ}^{7},\left(0.4,0.3\right)/{ℍ}^{8} \end{array}\right\}$
*q* ^3^	$\left\{\begin{array}{c} \left(0.4,0.3\right)/{ℍ}^{2},\left(0.5,0.4\right)/{ℍ}^{3},\left(0.6,0.3\right)/{ℍ}^{4} \end{array}\right\}$	*q* ^11^	$\left\{\begin{array}{c} \left(0.5,0.4\right)/{ℍ}^{2},\left(0.6,0.3\right)/{ℍ}^{4},\left(0.7,0.2\right)/{ℍ}^{6} \end{array}\right\}$
*q* ^4^	$\left\{\begin{array}{c} \left(0.6,0.2\right)/{ℍ}^{4},\left(0.7,0.3\right)/{ℍ}^{5},\left(0.8,0.1\right)/{ℍ}^{6} \end{array}\right\}$	*q* ^12^	$\left\{\begin{array}{c} \left(0.7,0.2\right)/{ℍ}^{2},\left(0.8,0.1\right)/{ℍ}^{3},\left(0.9,0.1\right)/{ℍ}^{6} \end{array}\right\}$
*q* ^5^	$\left\{\begin{array}{c} \left(0.2,0.1\right)/{ℍ}^{6},\left(0.1,0.2\right)/{ℍ}^{7},\left(0.4,0.3\right)/{ℍ}^{8} \end{array}\right\}$	*q* ^13^	$\left\{\begin{array}{c} \left(0.2,0.1\right)/{ℍ}^{3},\left(0.4,0.3\right)/{ℍ}^{5},\left(0.6,0.1\right)/{ℍ}^{7} \end{array}\right\}$
*q* ^6^	$\left\{\begin{array}{c} \left(0.4,0.2\right)/{ℍ}^{2},\left(0.3,0.4\right)/{ℍ}^{3},\left(0.4,0.5\right)/{ℍ}^{4} \end{array}\right\}$	*q* ^14^	$\left\{\begin{array}{c} \left(0.2,0.5\right)/{ℍ}^{1},\left(0.5,0.4\right)/{ℍ}^{3},\left(0.6,0.2\right)/{ℍ}^{5} \end{array}\right\}$
*q* ^7^	$\left\{\begin{array}{c} \left(0.2,0.3\right)/{ℍ}^{1},\left(0.3,0.4\right)/{ℍ}^{3},\left(0.4,0.3\right)/{ℍ}^{5} \end{array}\right\}$	*q* ^15^	$\left\{\begin{array}{c} \left(0.6,0.3\right)/{ℍ}^{5},\left(0.4,0.3\right)/{ℍ}^{7},\left(0.2,0.4\right)/{ℍ}^{8} \end{array}\right\}$
*q* ^8^	$\left\{\begin{array}{c} \left(0.1,0.4\right)/{ℍ}^{2},\left(0.3,0.5\right)/{ℍ}^{3},\left(0.5,0.4\right)/{ℍ}^{7} \end{array}\right\}$	*q* ^16^	$\left\{\begin{array}{c} \left(0.3,0.6\right)/{ℍ}^{4},\left(0.5,0.4\right)/{ℍ}^{5},\left(0.7,0.1\right)/{ℍ}^{6} \end{array}\right\}$

**Table 4:** Membership values *𝒯*_ℬ_^*D*^(ℍ^*i*^).

ℍ^*i*^	*𝒯*_ℬ_^*D*^(ℍ^*i*^)	ℍ^*i*^	*𝒯*_ℬ_^*D*^(ℍ^*i*^)	ℍ^*i*^	*𝒯*_ℬ_^*D*^(ℍ^*i*^)	ℍ^*i*^	*𝒯*_ℬ_^*D*^(ℍ^*i*^)
ℍ^1^	0.0406	ℍ^3^	0.1006	ℍ^5^	0.1006	ℍ^7^	0.0728
ℍ^2^	0.0950	ℍ^4^	0.0800	ℍ^6^	0.1676	ℍ^8^	0.0358

**Table 5:** Degrees of membership *𝒯*_*𝒟*_(*p*^*i*^).

*𝒯*_*𝒟*_(*p*^*i*^)	Degree	*𝒯*_*𝒟*_(*p*^*i*^)	Degree	*𝒯*_*𝒟*_(*p*^*i*^)	Degree	*𝒯*_*𝒟*_(*p*^*i*^)	Degree
*𝒯*_*𝒟*_(*p*^1^)	0.1	*𝒯*_*𝒟*_(*p*^5^)	0.5	*𝒯*_*𝒟*_(*p*^9^)	0.9	*𝒯*_*𝒟*_(*p*^13^)	0.35
*𝒯*_*𝒟*_(*p*^2^)	0.2	*𝒯*_*𝒟*_(*p*^6^)	0.6	*𝒯*_*𝒟*_(*p*^10^)	0.16	*𝒯*_*𝒟*_(*p*^14^)	0.75
*𝒯*_*𝒟*_(*p*^3^)	0.3	*𝒯*_*𝒟*_(*p*^7^)	0.7	*𝒯*_*𝒟*_(*p*^11^)	0.25	*𝒯*_*𝒟*_(*p*^15^)	0.65
*𝒯*_*𝒟*_(*p*^4^)	0.4	*𝒯*_*𝒟*_(*p*^8^)	0.8	*𝒯*_*𝒟*_(*p*^12^)	0.45	*𝒯*_*𝒟*_(*p*^16^)	0.85

**Table 6:** Approximate functions *ψ*_*𝒟*_(*p*^*i*^).

*p* ^*i*^	*ψ*_*𝒟*_(*p*^*i*^)	*p* ^*i*^	*ψ*_*𝒟*_(*p*^*i*^)
*p* ^1^	$\left\{\begin{array}{c} \left(0.2,0.1,0.2\right)/{ℍ}^{1},\left(0.3,0.2,0.1\right)/{ℍ}^{2} \end{array}\right\}$	*p* ^9^	$\left\{\begin{array}{c} \left(0.4,0.3,0.2\right)/{ℍ}^{2},\left(0.6,0.4,0.3\right)/{ℍ}^{7},\left(0.5,0.4,0.3\right)/{ℍ}^{8} \end{array}\right\}$
*p* ^2^	$\left\{\begin{array}{c} \left(0.1,0.2,0.1\right)/{ℍ}^{1},\left(0.5,0.4,0.3\right)/{ℍ}^{2},\left(0.1,0.4,0.3\right)/{ℍ}^{3} \end{array}\right\}$	*p* ^10^	$\left\{\begin{array}{c} \left(0.2,0.1,0.2\right)/{ℍ}^{6},\left(0.6,0.4,0.5\right)/{ℍ}^{7},\left(0.4,0.3,0.2\right)/{ℍ}^{8} \end{array}\right\}$
*p* ^3^	$\left\{\begin{array}{c} \left(0.4,0.3,0.1\right)/{ℍ}^{2},\left(0.5,0.4,0.3\right)/{ℍ}^{3},\left(0.6,0.3,0.2\right)/{ℍ}^{4} \end{array}\right\}$	*p* ^11^	$\left\{\begin{array}{c} \left(0.5,0.4,0.3\right)/{ℍ}^{2},\left(0.6,0.3,0.2\right)/{ℍ}^{4},\left(0.7,0.2,0.3\right)/{ℍ}^{6} \end{array}\right\}$
*p* ^4^	$\left\{\begin{array}{c} \left(0.6,0.2,0.3\right)/{ℍ}^{4},\left(0.7,0.3,0.4\right)/{ℍ}^{5},\left(0.8,0.1,0.4\right)/{ℍ}^{6} \end{array}\right\}$	*p* ^12^	$\left\{\begin{array}{c} \left(0.7,0.2,0.5\right)/{ℍ}^{2},\left(0.8,0.1,0.5\right)/{ℍ}^{3},\left(0.9,0.1,0.7\right)/{ℍ}^{6} \end{array}\right\}$
*p* ^5^	$\left\{\left(0.2,0.1,0.1\right)/{ℍ}^{6}\begin{array}{c} ,\left(0.1,0.2,0.1\right)/{ℍ}^{7},\left(0.4,0.3,0.1\right)/{ℍ}^{8} \end{array}\right\}$	*p* ^13^	$\left\{\begin{array}{c} \left(0.2,0.1,0.2\right)/{ℍ}^{3},\left(0.4,0.3,0.2\right)/{ℍ}^{5},\left(0.6,0.1,0.4\right)/{ℍ}^{7} \end{array}\right\}$
*p* ^6^	$\left\{\begin{array}{c} \left(0.4,0.2,0.3\right)/{ℍ}^{2},\left(0.3,0.4,0.3\right)/{ℍ}^{3},\left(0.4,0.5,0.3\right)/{ℍ}^{4} \end{array}\right\}$	*p* ^14^	$\left\{\begin{array}{c} \left(0.2,0.5,0.4\right)/{ℍ}^{1},\left(0.5,0.4,0.6\right)/{ℍ}^{3},\left(0.6,0.2,0.5\right)/{ℍ}^{5} \end{array}\right\}$
*p* ^7^	$\left\{\begin{array}{c} \left(0.2,0.3,0.4\right)/{ℍ}^{1},\left(0.3,0.4,0.4\right)/{ℍ}^{3},\left(0.4,0.3,0.4\right)/{ℍ}^{5} \end{array}\right\}$	*p* ^15^	$\left\{\begin{array}{c} \left(0.6,0.3,0.3\right)/{ℍ}^{5},\left(0.4,0.3,0.4\right)/{ℍ}^{7},\left(0.2,0.4,0.5\right)/{ℍ}^{8} \end{array}\right\}$
*p* ^8^	$\left\{\begin{array}{c} \left(0.1,0.4,0.3\right)/{ℍ}^{2},\left(0.3,0.5,0.6\right)/{ℍ}^{3},\left(0.5,0.4,0.7\right)/{ℍ}^{7} \end{array}\right\}$	*p* ^16^	$\left\{\begin{array}{c} \left(0.3,0.6,0.5\right)/{ℍ}^{4},\left(0.5,0.4,0.8\right)/{ℍ}^{5},\left(0.7,0.1,0.6\right)/{ℍ}^{6} \end{array}\right\}$

**Table 7:** Membership values *𝒯*_*𝒟*_^*D*^(ℍ^*i*^).

ℍ^*i*^	*𝒯*_*𝒟*_^*D*^(ℍ^*i*^)	ℍ^*i*^	*𝒯*_*𝒟*_^*D*^(ℍ^*i*^)	ℍ^*i*^	*𝒯*_*𝒟*_^*D*^(ℍ^*i*^)	ℍ^*i*^	*𝒯*_*𝒟*_^*D*^(ℍ^*i*^)
ℍ^1^	0.0431	ℍ^3^	0.1588	ℍ^5^	0.1656	ℍ^7^	0.1588
ℍ^2^	0.1825	ℍ^4^	0.1606	ℍ^6^	0.0964	ℍ^8^	0.1231

**Table 8:** Comparison with existing models under consideration of data given in Examples 2, 4, Definitions 7, and 18.

Authors	Structure	Domain parameterization	Range approximation	Type of approximate function	Ranking under *hs*-set environment
Çağman et al. [43]	fpfs-set	Fuzzy set parameterization	Fuzzy soft set	Single-argument	Inadequate
Deli and Çağman [44]	ifps-set	Intuitionistic fuzzy set parameterization	Fuzzy soft set	Single-argument	Inadequate
Joshi et al. [47]	ifpfs-set	Intuitionistic fuzzy set parameterization	Fuzzy soft set	Single-argument	Inadequate
Karaaslan [48]	ifpifs-set	Intuitionistic fuzzy set parameterization	Intuitionistic fuzzy soft set	Single-argument	Inadequate
Riaz and Hashmi [49]	fpfs-set	Fuzzy set parameterization	Fuzzy soft set	Single-argument	Inadequate
Zhu and Zhan [50]	fpfs-set	Fuzzy set parameterization	Fuzzy soft set	Single-argument	Inadequate
Proposed model	fpifhs-set	Fuzzy set parameterization	Intuitionistic fuzzy hypersoft set	Multiargument	*H*_6_ > *H*_3_=*H*_5_ > *H*_2_ > *H*_4_ > *H*_7_ > *H*_1_ > *H*_8_
Proposed model	fpnhs-set	Fuzzy set parameterization	Neutrosophic hypersoft set	Multiargument	*H*_2_ > *H*_5_ > *H*_4_ > *H*_3_=*H*_7_ > *H*_8_ > *H*_6_ > *H*_1_

**Table 9:** Comparison with existing models under appropriate features.

Authors	Structure	MD	NMD	ID	SAAF	MAAF
Çağman et al. [43]	fpfs-set	✔	×	×	✔	×
Deli and Çağman [44]	ifps-set	✔	✔	×	✔	×
Joshi et al. [47]	ifpfs-set	✔	✔	×	✔	×
Karaaslan [48]	ifpifs-set	✔	✔	×	✔	×
Riaz and Hashmi [49]	fpfs-set	✔	×	×	✔	×
Zhu and Zhan [50]	fpfs-set	✔	×	×	✔	×
Proposed model	fpifhs-set	✔	✔	×	✔	✔
Proposed model	fpnhs-set	✔	✔	✔	✔	✔

## Data Availability

No data were used to support this study.

## References

[1] Zadeh L. A. (1965). Fuzzy sets. *Information and Control*.

[2] Atanassov K. T. (1986). Intuitionistic fuzzy sets. *Fuzzy Sets and Systems*.

[3] Deli İ., Keleş M. A. (2021). Distance measures on trapezoidal fuzzy multi-numbers and application to multi-criteria decision-making problems. *Soft Computing*.

[4] Mahmood T., Ullah K., Khan Q., Jan N. (2019). An approach toward decision-making and medical diagnosis problems using the concept of spherical fuzzy sets. *Neural Computing & Applications*.

[5] Ünver M., Olgun M., Türkarslan E. (2022). Cosine and cotangent similarity measures based on choquet integral for spherical fuzzy sets and applications to pattern recognition. *Journal of Computational and Cognitive Engineering*.

[6] Wang L., Garg H. (2021). Algorithm for multiple attribute decision-making with interactive Archimedean norm operations under Pythagorean fuzzy uncertainty. *International Journal of Computational Intelligence Systems*.

[7] Smarandache F. (1998). *Neutrosophy, Neutrosophic Probability, Set, and Logic, Analytic Synthesis and Synthetic Analysis*.

[8] Molodtsov D. (1999). Soft set theory-first results. *Computers & Mathematics with Applications*.

[9] Çağman N., Enginoğlu S., Çitak F. (2011). Fuzzy soft set theory and its applications. *Iranian Journal of Fuzzy System*.

[10] Maji P. K., Biswas R., Roy A. R. (2001). Fuzzy soft sets. *Journal of Fuzzy Mathematics*.

[11] Çağman N., Karataş S., Karataş S. (2013). Intuitionistic fuzzy soft set theory and its decision making. *Journal of Intelligent and Fuzzy Systems*.

[12] Maji P. K., Biswas R., Roy A. R. (2001). Intuitionistic fuzzy soft sets. *Journal of Fuzzy Mathematics*.

[13] Maji P. K. (2013). Neutrosophic soft set. *Annals of Fuzzy Mathematics and Informatics*.

[14] Ali M. I., Feng F., Liu X., Min W. K., Shabir M. (2009). On some new operations in soft set theory. *Computers & Mathematics with Applications*.

[15] Li F. (2011). Notes on soft set operations. *ARPN Journal of systems and softwares*.

[16] Maji P. K., Biswas R., Roy A. R. (2003). Soft set theory. *Computers & Mathematics with Applications*.

[17] Pei D., Miao D. (2005). From soft set to information system. *International Conference of Granular Computing IEEE*.

[18] Sezgin A., Atagün A. O. (2011). On operations of soft sets. *Computers & Mathematics with Applications*.

[19] Babitha K. V., Sunil J. J. (2010). Soft set relations and functions. *Computers & Mathematics with Applications*.

[20] Babitha K. V., Sunil J. J. (2011). Transitive closures and orderings on soft sets. *Computers & Mathematics with Applications*.

[21] Broumi S., Deli I., Smarandache F. (2014). Neutrosophic parametrized soft set theory and its decision making. *International Frontier Science Letters*.

[22] Deli I. (2017). Interval-valued neutrosophic soft sets and its decision making. *International Journal of Machine Learning and Cybernetics*.

[23] Smarandache F. (2018). Extension of soft set of hypersoft set, and then to plithogenic hypersoft set. *Neutrosophic Sets and Systems*.

[24] Saeed M., Rahman A. U., Ahsan M., Smarandache F. (2021). An inclusive study on fundamentals of hypersoft set. *Theory and Application of Hypersoft Set*.

[25] Debnath S. (2021). Fuzzy hypersoft sets and its weightage operator for decision making. *Journal of Fuzzy Extension and Applications*.

[26] Deli İ. (2021). Hybrid set structures under uncertainly parameterized hyper soft sets. *Theory and Application of Hypersoft Set*.

[27] Kamacı H., Saqlain M. (2021). n-ary Fuzzy hypersoft expert sets. *Neutrosophic Sets and Systems*.

[28] Martin N., Smarandache F. (2020). Concentric plithogenic hypergraph based on plithogenic hypersoft sets–a novel outlook. *Neutrosophic Sets and Systems*.

[29] Martin N., Smarandache F., Broumi S. (2021). Covid-19 decision-making model using extended plithogenic hypersoft sets with dual dominant attributes. *International journal of neutrosophic science*.

[30] Rahman A. U., Saeed M., Smarandache F., Ahmad M. R. (2020). Development of hybrids of hypersoft set with complex fuzzy set, complex intuitionistic fuzzy set and complex neutrosophic set. *Neutrosophic Sets and Systems*.

[31] Rahman A. U., Saeed M., Smarandache F. (2020). Convex and concave hypersoft sets with some properties. *Neutrosophic Sets and Systems*.

[32] Rahman A. U., Saeed M., Dhital A. (2021). Decision making application based on neutrosophic parameterized hypersoft set theory. *Neutrosophic Sets and Systems*.

[33] Rahman A. U., Saeed M., Alodhaibi S., Abd El-Wahed Khalifa H. (2021). Decision making algorithmic approaches based on parameterization of neutrosophic set under hypersoft set environment with fuzzy, intuitionistic fuzzy and neutrosophic settings. *Computer Modeling in Engineering and Sciences*.

[34] Saeed M., Ahsan M., Saeed M. H., Mehmood A., Abdeljawad T. (2021). An application of neutrosophic hypersoft mapping to diagnose hepatitis and propose appropriate treatment. *IEEE Access*.

[35] Saeed M., Rahman A. U., Arshad M. (2021). A study on some operations and product of neutrosophic hypersoft graphs. *Journal of Applied Mathematics and Computing*.

[36] Saeed M., Ahsan M., Ur Rahman A., Saeed M. H., Mehmood A. (2021). An application of neutrosophic hypersoft mapping to diagnose brain tumor and propose appropriate treatment. *Journal of Intelligent and Fuzzy Systems*.

[37] Saqlain M., Moin S., Jafar N., Saeed M., Smarandache F. (2020). Aggregate operators of neutrosophic hypersoft sets. *Neutrosophic Sets and Systems*.

[38] Saqlain M., Riaz M., Saleem M. A., Yang M.-S. (2021). Distance and similarity measures for neutrosophic hypersoft set (nhss) with construction of nhss-topsis and applications. *IEEE Access*.

[39] Zulqarnain R. M., Xin X. L., Saqlain M., Smarandache F. (2020). Generalized aggregate operators on neutrosophic hypersoft set. *Neutrosophic Sets and Systems*.

[40] Adam F., Hassan N. (2014). Multi Q-fuzzy parameterized soft set and its application. *Journal of Intelligent and Fuzzy Systems*.

[41] Alkhazaleh S., Salleh A. R., Hassan N. (2011). Fuzzy parameterized interval-valued fuzzy soft set. *Applied Mathematical Sciences*.

[42] Aydın T., Enginoğlu S. (2021). Interval-valued intuitionistic fuzzy parameterized interval-valued intuitionistic fuzzy soft sets and their application in decision-making. *Journal of Ambient Intelligence and Humanized Computing*.

[43] Çağman N., Çitak F., Enginoğlu S. (2010). Fuzzy parameterized fuzzy soft set theory and its applications. *Turkish Journal of Fuzzy System*.

[44] Deli I., Çağman N. (2015). Intuitionistic fuzzy parameterized soft set theory and its decision making. *Applied Soft Computing*.

[45] Hassan N., Al-Qudah Y. (2019). Fuzzy parameterized complex multi-fuzzy soft set. *Journal of Physics: Conference Series*.

[46] Hazaymeh A., Abdullah I. B., Balkhi Z., Ibrahim R. (2012). Fuzzy parameterized fuzzy soft expert set. *Applied Mathematical Sciences*.

[47] Joshi B. P., Kumar A., Singh A., Bhatt P. K., Bharti B. K. (2018). Intuitionistic fuzzy parameterized fuzzy soft set theory and its application. *Journal of Intelligent and Fuzzy Systems*.

[48] Karaaslan F. (2016). Intuitionistic fuzzy parameterized intuitionistic fuzzy soft sets with applications in decision making. *Annals of Fuzzy Mathematics and Informatics*.

[49] Riaz M., Hashmi M. R. (2016). Certain applications of fuzzy parameterized fuzzy soft sets in decision-making problems. *International Journal of Algebra and Statistics*.

[50] Zhu K., Zhan J. (2016). Fuzzy parameterized fuzzy soft sets and decision making. *International Journal of Machine Learning and Cybernetics*.

[51] Musa S. Y., Asaad B. A. (2021). Bipolar hypersoft sets. *Mathematics*.

